# Rational Design of EV-Mimicking Nanoparticles with Polarity-Based Recognition Potential for Advanced Nanocarrier Development

**DOI:** 10.1021/acsanm.5c01459

**Published:** 2025-06-23

**Authors:** Giada Rosso, Stijn M.A. Van Veen, María Sancho-Albero, Giulia Tamboia, Charly Empereur-Mot, Claudio Perego, Marije E. Kuipers, Bianca Dumontel, Alessandro Ajó, Esther N. Nolte-’t Hoen, Giovanni M. Pavan, Luisa De Cola, Lorenzo Albertazzi, Valentina Cauda

**Affiliations:** † Department of Applied Science and Technology, 151697 Politecnico di Torino, Turin 10129, Italy; ‡ Institute of Complex Molecular Systems, 3169 Technische Universiteit Eindhoven, Eindhoven 5600 MB, Netherlands; § Department of Biochemistry and Molecular Pharmacology, 9304 Istituto di Ricerche Farmacologiche Mario Negri IRCCS, Milan 20157, Italy; ∥ Department of Innovative Technologies, 30446 University of Applied Science and Arts of Southern Switzerland, Lugano 6962, Switzerland; ⊥ Department of Biomolecular Health Sciences, 8125 Utrecht University, Utrecht 3584 CH, Netherlands; # Department of Pharmaceutical Science, DISFARM, Università degli Studi di Milano, Milan 20133, Italy; ∇ Department of Chemistry, Biology and Biotechnology, Università di Perugia, Perugia 06123, Italy

**Keywords:** biomimetic, nanomedicine, artificial, extracellular vesicles, lipid nanoparticles, super-resolutionimaging

## Abstract

Extracellular vesicles (EVs) are natural carriers that are essential for intracellular communication, delivering biomolecules with high efficiency and selectivity. Their application in a clinical setting has been limited, however, due to their complexity and heterogeneity, which hamper standardization in isolation procedures. A solution could be to engineer synthetic nanoparticles that are able to mimic the natural EV structure and function, which would lead to innovative therapeutic nanoplatforms with key advantages over traditional synthetic nanoparticles in terms of toxicity and efficacy. Here, we report an approach to designing, synthesizing, and characterizing lipid-coated nanoparticles engineered to replicate key biophysical surface properties of EVs relevant to cellular recognition and biointerface interactions. Three different lipidic mixtures were designed based on lipidomic data of prostate cancer-derived EVs, taking into consideration the mass percentage of both the lipid families and the fatty acids. Furthermore, breakable organosilica nanocapsules were employed as a functional core and coated with the lipidic mixtures to form eventual EV-mimicking nanocarriers (EV Mimics). Computational modeling of the lipid bilayer was employed to further optimize the lipid coverage of the organosilica nanocapsules. In addition to conventional characterization techniques, which assessed the matching of size and surface charge of EV Mimics and natural EVs, we used advanced single-particle characterization techniques, such as high-resolution flow cytometry and super-resolution microscopy, to assess coating efficacy, size distribution, and lipid polarity—a key parameter in cellular uptake and membrane interaction of EV Mimics. This multidisciplinary approach led to the discovery of a formulation (called “CE Mimic 3”, composed of Chol/SM/PE/PC/PS with respective mass ratios of 30/16.1/12.9/20.9/20.1) that closely reproduces the size, charge, lipid coating, and polarity of natural EVs, thus laying the groundwork for the development of EV-mimetic nanoplatforms for biomedical applications such as targeted delivery or biosensing.

## Graphical abstract

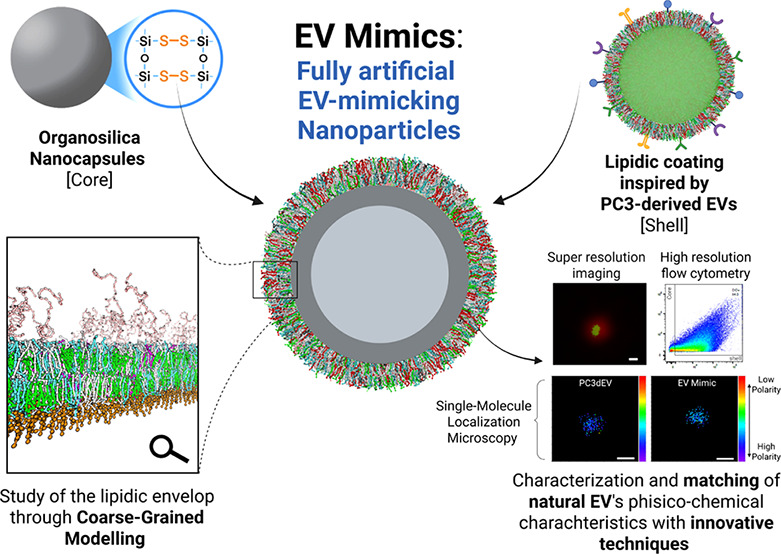

## Introduction

Nanomedicine has recently gained more traction after the success of liposomal mRNA vaccines.^1^ Nanoparticles, and especially liposomes, present a number of advantages. They are easily internalized by cells due to their small size,^2^ can act as carriers for otherwise poorly soluble or hydrophobic drugs,^3^ facilitate controlled release of cargo,^4^ and can be chemically modified through conjugation with various ligands.^5^ Despite these advantages, translation to the clinic has had a low success rate, with only approximately 30 nanomedicine formulations approved by the EMA in nearly 30 years.^6,7^ There are many factors at play that have led to this shortcoming. These include off-target accumulation, unexpected toxicity effects, and the intrinsic heterogeneity of nanoparticle formulations.^8^

Along with synthetic nanomedicine, extracellular vesicles (EVs) have also gained more attention as natural, cell-derived nanoparticles.^9,10^ EVs are naturally secreted by cells and feature a double phospholipidic layer containing membrane proteins, similar to cell membranes. The vesicle lumen contains various proteins, metabolites, and nucleic acids. They act as biological nanocarriers and have been shown to play a key role in intercellular communication by transporting functional molecules such as bioactive lipids, proteins, or RNA to target cells.^11–14^ In particular, EVs derived from cancer cells exhibit high levels of tropism, i.e., the ability to target specific cell or tissue types, and may even cross cellular barriers,^15,16^ such as the blood–brain barrier, to reach distant tissues or organs in a highly selective way. These features are fundamental requirements for an ideal drug delivery system and make EVs extremely interesting for therapeutic applications.^17–19^ In recent years, a vast field of research has emerged, where efforts are being made to engineer and load EVs with therapeutic agents to treat a broad spectrum of pathologies.^13,17–19^ However, there are currently no EV-based products available on the market, as the use of EVs in the clinic is hampered by costly manufacturing, lack of standardization, and low loading efficiency—leading to poor reproducibility in the synthesis of EVs and a large degree of heterogeneity within the EV population.^7,19^ To overcome these limitations, an alternative strategy could be to develop fully artificial nanoparticles that mimic the properties of natural EVs while enabling scalable production, cargo loading, and tailored functionalization. As we have previously reported,^20^ attempts have already been made to generate EV-mimicking liposomes.^21,22^ A hybrid strategy has also been explored, in which liposomes are fused with EVs through extrusion.^23^ These hybrid particles can exhibit intriguing functionalities, such as the ability to activate endothelial signaling pathways. However, this approach lacks control over the composition and specificity of the final product, as it relies on the random incorporation of components from a heterogeneous EV population.

In this work, we produced and characterized artificial nanocarriers (referred to hereafter as “EV Mimics”), combining a breakable organosilica core with a lipid bilayer that aims to mimic the EV’s physicochemical properties critical for cellular interaction, such as size, surface charge, lipid composition, and polarity. These artificial EV Mimics have several key advantages compared with their natural counterparts. They are more easily produced in bulk, and the procedure is simple to standardize. Moreover, the organosilica nanocapsules (NCs) can be synthesized enclosing their cargo (either drugs or proteins),^24^ which facilitates high loading capacities that are difficult to achieve with just liposomes or isolated natural EVs. The organosilica core is also breakable, which allows for a stimuli-responsive release of therapeutic or imaging cargo, as previously reported.^25^ As a proof of concept, we attempt to mimic EVs from the prostate cancer cell line PC3, as they have shown a strong tropism toward bone tissue, providing a clear clinical target for the future.^26^

The EVs produced by a particular cell line exhibit distinct characteristics reflecting the state of the cell they originate from (i.e., specific structure and composition of the lipid membrane, its microviscosity, embedded proteins, etc.).^27,28^ When trying to mimic their properties, it is important to also consider the physicochemical properties of their molecular components. Specifically, the lipid composition of EV membranes may influence properties such as membrane polarity, fluidity, and surface charge—all factors that could impact cellular recognition, uptake, and tropism. The lipid composition of the EV membranes has a strong effect on the physical properties of EVs, which could be a key factor in their functionality. Therefore, various lipid compositions were prepared based on previously reported lipidomic data of PC3 EVs.^29^ To gain detailed insights into the EV Mimic formulations, we combined conventional characterization techniques—including assessments of size distribution, *Z*-potential, and morphology—with molecular dynamics simulations to estimate lipid interactions, and single-particle techniques to examine population heterogeneity. High-resolution flow cytometry was used to evaluate the coating efficiency of the Mimic formulations, while super-resolution single-molecule localization microscopy (SMLM) was combined with a solvatochromic dye to probe and compare the membrane polarity of natural EVs and the synthesized EV Mimics with nanometer resolution. This polarity profiling provides a unique window into the nanoscale membrane environment, offering a potential proxy for how these synthetic carriers may be perceived by biological systems. While biology and nanomedicine are incredibly complex, the multiparametric characterization shown in this work lays essential groundwork for the synthesis of artificial vesicles that mimic the structural and physical features of naturally occurring EVs, thus paving the way for drug delivery applications.

## Experimental Section

### Nanocapsules Synthesis and Characterization

The stimuli-responsive organosilica nanocapsules (NCs) were synthesized using the Stöber process in a W/O microemulsion, following a previously published protocol.^24^ Briefly, 1.77 mL of TRITON X-100, 7.5 mL of cyclohexane, and 1.8 mL of *n*-hexanol were mixed in a 50 mL round-bottom flask and stirred on a magnetic stirrer for 30 min. Separately, 600 μL of dH2O were mixed with 40 μL of tetraethyl orthosilicate (TEOS) and 60 μL of bis­[3-(triethoxysilyl)­propyl]­disulfide (BTDS). After stirring, this mixture was added to the organic solution. The hydrolysis of TEOS was initiated by adding 50 μL of 28% aq. NH_3_, and the mixture was stirred at room temperature overnight. Subsequently, 20 mL of pure acetone was added to precipitate the NCs, and the material was recovered by centrifugation (35000 *g*, 30 min at room temperature). The NCs pellet was redispersed in ethanol and washed again before being suspended in dH2O and stored until use.

Once the NCs were synthesized, they were further functionalized with 2,2-dimethoxy-1,6-diaza-2-silacyclooctane (Gelest, United States). Specifically, 10% of the positively charged molecules were incubated with the naked NCs in toluene overnight at room temperature. Subsequently, the functionalized NCs were washed twice by centrifugation with ethanol and stored at 4 °C until they were used.

The resulting NCs and positively charged NCs were characterized using transmission electron microscopy (TEM; Talos L120C from FEI). For TEM analysis, samples were deposited onto a copper grid. The size distribution and surface charge of the nanoparticles were obtained using dynamic light scattering (DLS) instrument. The DLS measurements were performed on dispersions of NCs in dH_2_O (pH 7) using a Zetasizer Ultra instrument equipped with Multiangle Dynamic Light Scattering (MALDLS) technology from Malvern Panalytical and the ZS Xplorer software. Fourier transform infrared (FTIR) spectra were recorded by using a Shimadzu IRAffinity-1 spectrometer. The transmittance spectrum was collected using a spectral resolution of 1 cm^–1^, accumulating 64 scans from 600 to 4000 cm^–1^. TGA was conducted on a Netzsch model STA 449 Fi Jupiter instrument. The samples (0.5–3 mg) were kept at 100 °C for 30 min for stabilization, then heated from 130 to 800 °C at a speed of 10 °C min^–1^, before being held at this temperature for a further 30 min at the end of the measurement. The analysis was carried out under a 20 mL min^–1^ airflow.

### Development of EV Mimics Formulations

In order to mimic the lipidic composition of PC3-derived EVs (PC3dEVs), the work by Ferreri et al.,^29^ in which the lipidomic profile of PC3dEVs is presented (and reported here in Tables S1 and S2), was used as a reference for the design of the three EV-mimicking formulations, taking care to respect the same proportion of the main phospholipid families, i.e., Sphingomyelins (SM), Phosphatidylethanolamine (PE), Phosphatidylserine (PS), Phosphatidylcholine (PC), and cholesterol, as well as the ratio between the most abundant species of fatty acids in the natural composition. Table 1 reports the mass percentages of the lipidic components designed for Formulation 3C, Mimic 1, 2, and 3, in comparison to the natural PC3dEVs .

**Table 1:** EV-Mimicking Artificial Lipidic Compositions Compared to 3C Formulation and PC3dEVs’ Natural Composition in Terms of the Mass Percentage of Each Lipid Species

Lipid Families in PC3dEVs	Commercial products	Mass percentage ± SEM in PC3dEVs	3C	Mimic 1	Mimic 2	Mimic 3
Chol	Cholesterol	12.6 ± 2.2%	23.6%	13.0%	13.0%	13.0%
SM	16:0 SM (d18:1/16:0)	20.2 ± 11.8%	/	20.0%	20.0%	20.0%
PE	DSPE-PEG(2000) Amine	16.1 ± 4.9%	6.6%	16.0%	16.0%	10.0%
	18:2 PE	/	/	/	/	6.0%
PC	DOPC (18:1 (Δ9-Cis) PC)	26.1 ± 7.4%	12.5%	26.0%	26.0%	26.0%
PS	16:0 PS	25.0 ± 9.6%	/	/	25.0%	25.0%
/	18:1 PA	/	57.3%	25.0%	/	/

To prepare the formulations, cholesterol (chloroform solution) was purchased from Sigma-Aldrich, and phospholipids were purchased from Avanti Polar Lipids Inc.: 16:0 SM (d18:1/16:0) (*N*-palmitoyl-d-erythro-sphingosylphosphorylcholine); DSPE-PEG(2000) Amine (1,2-distearoyl-*sn*-glycero-3-phosphoethanolamine-*N*-[amino­(polyethylene glycol)-2000] (ammonium salt)); 18:2 PE (1,2-dilinoleoyl-*sn*-glycero-3-phosphoethanolamine); DOPC (18:1 (Δ9-Cis) PC (DOPC), 1,2-dioleoyl-*sn*-glycero-3-phosphocholine, chloroform solution); 16:0 PS (1,2-dipalmitoyl-*sn*-glycero-3-phospho-l-serine (sodium salt)); and DOPA (18:1 PA, 1,2-dioleoyl-*sn*-glycero-3-phosphate (sodium salt), chloroform solution).

To further optimize the lipid coating, another version of the Mimic formulations was tested, including a higher amount of cholesterol. The mass percentage of cholesterol was increased to 30%, and the other lipids in the formulations were rebalanced accordingly, maintaining the same proportions of SM, PE, PC, and PS. The resulting compositions are reported in Table 2.

**Table 2:** Mass Percentage of Lipid Species in Cholesterol-Enriched (CE) EV-Mimicking Artificial Lipidic Compositions

Lipid Families in PC3dEVs	Commercial products	CE Mimic 1	CE Mimic 2	CE Mimic 3
Chol	Cholesterol	30.0%	30.0%	30.0%
SM	16:0 SM (d18:1/16:0)	16.1%	16.1%	16.1%
PE	DSPE-PEG(2000) Amine	12.9%	12.9%	8.0%
	18:2 PE	/	/	4.8%
PC	DOPC (18:1 (Δ9-Cis) PC)	20.9%	20.9%	20.9%
PS	16:0 PS	/	20.1%	20.1%
/	18:1 PA	20.1%	/	/

### Assembly and Characterization of EV Mimics

NCs@NH_2_ were covered by the above-described lipid formulations using the solvent exchange method,^30^ developed, already tested, and optimized by some of us on another kind of organosilica nanostructure.^25^ In brief, the various lipidic components were mixed together in a glass vial in the previously established proportions and allowed to dry overnight under vacuum conditions. Successively, the dried lipid film was rehydrated by adding a 2:3 (v/v) ethanol:water solution, achieving a final concentration of lipids equal to 3 mg/mL. NCs were also dispersed in the same solution, and the lipid mixture was added, observing a 2:1 NCs:lipids mass ratio, and the obtained suspension was sonicated for 3 min in an ultrasound (US) bath (59 kHz, Branson 3800 CPXH, Branson Ultrasonics Corporation, Brookfield, CT, USA). Afterward, bdH_2_O was quickly added to the mixture to exceed 70% of the solution’s volume. This sudden change in the solution’s proportions drives the self-assembly of the phospholipids, which orient their hydrophobic heads toward the solution and the surface of NCs@NH_2_, thus forming a lipid bilayer around them. To further homogenize the suspension of lipid-coated NCs, another sonication step in the US bath (5 min) was performed.

To characterize the size distribution and colloidal stability of the coated nanoparticles, DLS and *Z*-potential (Zetasizer Nano ZS90 from Malvern Instruments), as well as nanoparticle tracking analysis (NTA, NanoSight NS300 from Malvern Panalytical), were performed in deionized water at room temperature. All measurements were conducted 3 times and then averaged. For NTA analysis, three 60 s videos of each sample flowing through the instrument chamber were recorded and analyzed with the NTA 3.4 software from Malvern Panalytical.

Transmission Electron Microscopy (TEM) was performed to determine the morphology and structure of the materials, whereas Scanning-Transmission Electron Microscopy (STEM) was performed to determine the composition of the samples. Both techniques were carried out using a Tecnai F30 microscope (FEI Company) at a working voltage of 300 kV. TEM images were obtained with a coupled CCD camera (Gatan). Regarding STEM, High-Angle Annular Dark Field (STEM-HAADF) images were obtained with a HAADF detector (Fischione). Also, in order to analyze the chemical composition of the materials and corroborate the presence of Si in the EV Mimics, X-ray Energy Dispersive Spectra (EDS) were obtained with an EDAX detector. An 8 μL drop of each sample was placed on Parafilm on a Petri dish. Freshly glow-discharged (30 s, 15 mA) carbon-coated 200-mesh copper grids (Agar Scientific Supplies) were incubated for 5 min on the sample drops. Excess liquid was removed by briefly contacting the edge of the grid with filter paper, and the grids were allowed to air-dry.

For the Cryo-TEM analysis, the morphology of the lipid-coated and naked NPs was analyzed by transmission electron microscopy (TEM) using a TECNAI G2 20 SUPER TWIN (FEI), operating at an accelerating voltage of 200 kV in bright-field image mode. The samples were diluted and then dried using a UV lamp. The preparation of the cryo-TEM samples first involved a vitrification procedure on an FEI Vitrobot Mark IV (Eindhoven, The Netherlands). One drop of the sample solution (∼3 μL) was deposited on a copper grid (300 mesh Quantifoil R2/2, hydrophilized by glow-discharge treatment just prior to use) within the environmental chamber of the Vitrobot, and the excess liquid was blotted away. The sample was shot into melting (liquid) ethane and transferred through a 655 Turbo Pumping Station (Gatan, France) to a 626 DH Single Tilt Cryo Holder (Gatan, France), where it was maintained below −170 °C (liquid nitrogen temperature). The sample was examined in the TECNAI G2 20 TWIN (FEI) previously mentioned, operating at an accelerating voltage of 200 kV in brightfield. TEM images were acquired with a Veleta 2K x 2K CCD camera. The Cryo-TEM characterization of the naked and coated particles was performed by the Synthesis of Nanoparticles Unit (UNIT 9) of the ICTS “Nanbiosis” at the Institute of Nanoscience and Materials of Aragon (INMA)­nUniversidad de Zaragoza.

To fluorescently label the NCs, Atto647-NHS or Atto488-NHS ester (Thermo Fisher, Waltham, MA, USA) was added (4 μg/mg of NCs) to the ethanolic suspension of NCs@NH_2_ and stirred in the dark overnight. The material was then washed by centrifugation (14000 *g*, 30 min) through multiple cycles until the supernatant became colorless.

### Optimization of NCs/Lipids Mass Ratio

NCs dimensions were estimated from TEM images: NCs diameter and wall thickness were calculated with ImageJ software; a distribution of diameters was obtained and the frequency was calculated using bins of 5 nm. From the above, the volume of a single NC was calculated, considering it as a perfect hollow sphere. To obtain the weight of a single NC, the volume value was multiplied by the silica density of 1.87 g/m^3^, reported in the literature for silica nanoparticles obtained with similar synthesis processes.^31^ These calculations were done for each NC diameter ranging from 45 and 125 nm, with a step of 5 nm. Instead, the weight of the lipid bilayer was evaluated by estimating the amount of lipids required for the coating of a sphere of a given diameter. To this purpose, we used the CHARMM-GUI MARTINI vesicle maker tool,^32–34^ which employs the structural parameters of MARTINI lipid and cholesterol models to generate spherical vesicles of a given inner radius. Successively, the ratio between the obtained weight estimation of NCs and lipid bilayers was calculated for each considered diameter. And finally, the obtained ratios were weighted by multiplying the value of the ratio by the frequency percentage of the diameter in the NCs’ size distribution, obtaining a comprehensive value for the NCs/lipids mass ratio.

### Cell Culture and Isolation of Natural PC3-Derived Extracellular Vesicles

PC3 cells were cultured in RPMI 1640 GlutaMAX (Gibco, Thermo Fisher Scientific, Waltham, MA, USA) supplemented with 10% FBS (Serana, Pessin, Germany) and penicillin–streptomycin (pen/strep, Gibco) in T175 flasks (Greiner Bio-One, Kremsmünster, Austria). When the cells reached 85% confluency, the medium was replaced by RPMI 1640 GlutaMAX supplemented with 10% EV-depleted FBS + pen/strep. For this, a 30% FBS in RPMI was depleted of EVs by >16 h of centrifugation at 28,000 rpm (∼100,000 *g*) in an SW32 Ti rotor and L90K ultracentrifuge (Beckman Coulter, Brea, CA, USA) at 4 °C. After centrifugation, the top 25 mL of each tube was collected using a serological pipette, filtered through a 0.2 μm filter, aliquoted, and stored at −20 °C. Medium for EV isolation was collected from the PC3 cells after 14–16 h of culture at 37 °C and 5% CO_2_. The culture medium was centrifuged twice at 200 *g* followed by two centrifugation steps at 500 *g*, all for 10 min each, and the supernatant was decanted into a new tube after each step. All centrifugation and following ultracentrifugation steps were done at 4 °C. The final 500 *g* supernatant was stored at −80 °C. For each EV isolation, 90 mL of 500 *g* supernatant was thawed overnight at 4 °C and then centrifuged for 30 min at 8,900 rpm (∼10,000 *g*) in an SW32 Ti rotor and L90K ultracentrifuge. All ultracentrifuge tubes used were thin-wall polypropylene. The 10,000 *g* supernatant was transferred to new tubes and centrifuged for 65 min at 28,000 rpm (∼100,000 *g*, SW32 Ti rotor, L90K centrifuge). The 100,000 *g* supernatants were removed until the conical part of the tube. The 100,000 *g* pellets were resuspended in the remaining supernatant and pooled into one SW40 Ti tube. The tube was topped up with 100,000 *g* supernatant and centrifuged for 65 min at 28,000 rpm (∼100,000 *g*) in an SW40 Ti rotor and XPN ultracentrifuge (Beckman Coulter). The final 100,000 *g* supernatant was removed, and the EV-enriched pellet was resuspended in 200 μL PBS + 0.2% BSA (made from a 5% BSA (Sigma-Aldrich, Saint Louis, MO, USA) in PBS stock that was depleted of particles by overnight ultracentrifugation, similar to the 30% FBS) and transferred to an SW60 tube. The resuspended EV pellet was gently mixed with 970 μL of 60% iodixanol (OptiPrep, Stemcell Technologies, Vancouver, Canada), after which the following iodixanol layers were built on top: 485 μL of 40%, 485 μL of 30%, and 1746 μL of 10%. The different iodixanol densities were made by mixing 50% (made from 60% iodixanol, 10× PBS, and sterile MQ water) and sterile PBS (Gibco). The density gradient was centrifuged for >16 h at 43,000 rpm (∼190,000 *g*) in an SW60 Ti rotor, with acceleration and deceleration settings of 9, in an XPN ultracentrifuge. From the gradient, 12 fractions of 324 μL were collected from top to bottom. Fractions 6 and 7 (where fraction 1 is the bottom and fraction 12 is the top) (with densities of 1.10–1.08 g/mL) were pooled into one SW40 tube and mixed with 11 mL of PBS + 0.1% BSA (particle-depleted). The purified EVs were pelleted for 65 min at 39,000 rpm (∼190,000 *g*) in an SW40 Ti rotor and XPN centrifuge. The supernatant was removed as before, and the final EV pellet was resuspended in 50 μL PBS + 0.1% BSA, aliquoted, and stored at −80 °C until imaging. Each aliquot was thawed only once.

## High-Resolution Flow Cytometry

NCs were labeled with Atto488-NHS ester, as described above. After lipid coating, the lipids were labeled by adding 5 μL of the lipophilic dye DiD (ThermoFisher, resuspended 1 mg/mL in dimethyl sulfoxide). EV Mimics were incubated at 37 °C for 15 min and then centrifuged at 5000g for 10 min to remove excess dye. The pellets were resuspended in water and diluted 1:1,000 in PBS (Gibco) before being measured on an Aurora spectral flow cytometer with an Enhanced Small Particle detector (Cytek). Each sample was measured with a B2 (peak detector for Atto488) threshold of 1200, which was set on unstained NCs to minimize background signals. Raw .fcs files were analyzed in FlowJo (version 10.1).

### Slide Preparation for Fluorescence Microscopy Imaging

Coverslips #1.5 were placed in an ultrasonic bath with fresh methanol for 15 min. Afterward, they were dried with nitrogen and underwent plasma treatment for 1 min (Openair FG 5001-Plasma Generator). To prepare a flow channel, two strips of double-sided tape were placed on a glass slide with the cleaned coverslip on top to form a chamber. For the EV samples, capture was aided by a mixture of primary antibodies containing 0.1 mg/mL of anti-CD9 (MAB-1880–100, R&D Systems), anti-CD63 (MAB5048, R&D Systems), and anti-CD81 (MAB4615, R&D Systems), which was flowed into the chamber and incubated for 30 min. Particles, whether they were EVs or EV Mimics, were flowed into the chamber and incubated for 30 min. Free particles were then washed away with either PBS or PBS containing 0.1% BSA in the case of EVs.

Nile Red stock solutions of 100 μM were prepared by dissolving Nile Red in high-purity dimethyl sulfoxide (DMSO) and then further diluting it into a PBS buffer. For each measurement, fresh dilutions of 5 nM Nile Red were prepared and added to the imaging chamber before closing it off with nail polish.

### Single-Molecule Localization Microscopy Imaging

Colocalization measurements of the Atto647-NHS ester-labeled nanoparticles and Nile Red localizations were obtained with an Oxford Nanoimager microscope (ONI, Oxford, UK).

The imaging was carried out in total internal reflection fluorescence (TIRF) configuration with a 100×, 1.4 NA oil-immersion objective. Images were acquired on a 428 × 428 pixel area, with a corresponding pixel size of 0.117 μm on an sCMOS camera. They were passed through a beam splitter to separate the two channels. To prevent cross-bleeding, images were recorded with an exposure time of 50 ms by first illuminating the nanoparticles for 3000 frames using a 640 nm laser at 3 mW, and subsequently recording the Nile Red signal for 10000 frames using a 561/532 nm laser at 30 mW. Before each measurement, a channel mapping calibration was performed using TetraSpeck beads (T7279, Invitrogen) to ensure proper colocalization.

Localization data were obtained through the in-built Gaussian fitting function in the ONI; then, it was drift-corrected and density-filtered in THUNDERSTORM.^35^ The resulting output was analyzed in nanoFeatures,^35^ which clustered and filtered particles to obtain parameters such as size and aspect ratio. The Nile Red localization data were overlaid with the diffraction-limited Atto647 signal of the nanocapsules. The percentage of Atto647-labeled particles that also showed a Nile Red signal was then determined manually.

### Spectral PAINT Imaging

The spectral Nile Red PAINT measurements were performed on an inverted optical microscope (Nikon Ti2) with a 70 grooves/mm grating (#46–068, Edmund Optics) placed in the emission path. The imaging was carried out in TIRF, illuminated with a 30 mW, 532 nm, continuous fiber-coupled laser source (FP1280764, Coherent OBIS). The excitation was directed through a dichroic (ZET 532/10X, Chroma) and a 100×, 1.49 NA oil-immersion objective (Nikon Apo TIRF 100× Oil). The emission passed through a mechanical slit (VA100C, Thorlabs), a long-pass filter (ET542LP, Chroma Technology USA), and a notch filter (NF533–17, Thorlabs) before reaching the previously mentioned grating and being projected onto an EMCCD camera (Andor DU-888 X-9414) with 250 multiplication gain, 50 ms exposure time, and 90 nm pixel size. The emission light was split 41% into the zeroth order and 32% into the first order according to the manufacturer. By adjusting the grating-to-camera distance and using an argon lamp (HG-2 Mercury Argon Calibration Light Source, Ocean Optics), we calibrated the dispersion versus wavelength.

Analysis is done in FIJI using THUNDERSTORM^35^ and RAINBOWSTORM.^36^ The TIFF file is loaded and separated into the spatial and spectral domains. The spatial image is then processed in THUNDERSTORM to obtain localization data. The background is removed from the spectral image, which is then used to identify and calculate the full spectra for each localization. To ensure good data quality, spectra with high uncertainty (>40 nm) and/or low spectral photon counts (<300) are filtered out. These data are then exported and postprocessed in THUNDERSTORM to do drift correction and density filtering as needed. Finally, a custom MATLAB script is used to do Gaussian peak fitting for each localization’s spectroscopic field, and the localizations are clustered using a mean shift clustering algorithm to correlate the data per particle. The script outputs both the mean and standard deviation of the bright times, dark times, centroid wavelengths, peak wavelengths, number of events, spatial photons, and spectral photons for each validated cluster. This gives information on the spectral properties of the whole population and individual particles.

### Spectral PAINT Calibration

The spectral setup was calibrated using an argon-based spectral calibration lamp (Ocean Optics HG-2), which shows lines at various wavelengths. The pixel distance to wavelength was calibrated for lines at 435, 586, and 763 nm. This was then fitted to obtain the relation between spatial-to-spectral distance and emission wavelength in nm.

### Molecular Simulations of EV Mimics Formulations

The molecular models for formulations CE Mimic 1, 2, and 3 were constructed using the recent MARTINI 3 coarse-grained (CG) force field,^37,57^ which provides improved lipid and cholesterol^38^ representations. Each simulation box contained a bilayer section of about 14.5 nm × 14.5 nm along the *x* and *y* axes, surrounded by a layer of MARTINI water of about 8 nm on each side along the *z* axis. Periodic boundary conditions (PBC) are imposed so that the opposite ends of the bilayer are continuously connected. Each bilayer section contains approximately 500 lipids and 500 cholesterol molecules (see Table S10 for a detailed description of the molecular content of all simulated systems). The bilayer models were constructed using the CHARMM-GUI MARTINI membrane builder.^34,39^

The organosilica surface model is constructed by placing MARTINI beads on a planar two-dimensional lattice with *x* and *y* spacings of 0.58 nm, so that they form an array of ligands having a density of 2.9 groups per nm^2^. This first layer of beads, representing the interfacial organosilica that reacted with 2,2-dimethoxy-1,6-diaza-2-silacyclooctane (DMDASCP), is restrained to the lattice sites using harmonic potentials with force constants k = 5000 kJ mol^–1^ nm^–2^. The DMDASCP groups are parameterized compatibly with the MARTINI 3 force field,^37,57^ using an N3d bead for representing the dimethoxysilicon moieties, a P3 bead for the secondary amine, and a charged (+1) SQ1 bead for the primary amine. This model allows representing the structure of the interface between water and the organosilica surface^40^ after the ring-opening click reaction of DMDASCP reagents.^41^ Furthermore, a harmonic restraining wall potential with a force constant *k* = 1000 kJ mol^–1^·nm^–2^ is employed to avoid the permeation of water and ions from the upper to the lower side of the surface, so that the lattice of N3d beads effectively represents the demarcation between bulk organosilica and the organosilica–DMDASCP–lipids interface. The starting configuration of the organosilica-supported lipid bilayer systems (”Supported”) was prepared by stacking the organosilica–DMDASCP surface model onto the lipid bilayer systems (”Free”), such that they are in direct contact before the minimization and equilibration procedures (i.e., many beads of the surface and the stacked lipid bilayer are distant by 0–0.5 nm). The total charge of the different EV mimic formulations, considered together with the charge of the functionalized surface when relevant, is neutralized by the addition of Na^+^ and Cl^–^ ions. In the organosilica-supported systems, neutralizing ions are inserted between the lipid bilayer and the surface, such that the models represent electrostatic equilibrium.

The MD simulations were performed using GROMACS^42^ (version 2021.4) using a 20 fs integration time step in the production stages (employed for analyses), which is standard in MARTINI simulations.^43^ We simulated the systems in the NPT ensemble (i.e., constant number of particles, pressure, and temperature), maintaining the temperature via the v-rescale^44^ algorithm (coupling constant set to 1 ps) and the pressure via the c-rescale^45^ algorithm, using a semi-isotropic scheme (coupling constant set to 4 ps and compressibility set to 3 × 10^–4^ bar^–1^). For each system, the CG-MD simulation protocol included a preliminary soft-core minimization stage (useful for handling steric clashes in the construction of the organosilica-supported systems), followed by 5000 steps of minimization using the steepest descent integrator and an equilibration stage of 2 μs using a 20 fs integration time step, during which the 310 K equilibrium temperature was reached. The equilibration stage also demonstrated the stability of the assemblies in organosilica-supported systems. The following production stage is 20 μs for each of the systems, which allowed us to obtain accurate sampling for the purpose of this study.

The bilayer thickness *D*_*HH*_ and APL are estimated by measuring every 10 ns over the 20 μs of production runs and computing the average and standard error. The area compressibility modulus is obtained from the APL measurement according to the relation $K_{A}=k_{B}T\frac{\mathrm{APL}}{\sigma_{\mathrm{APL}}}$ , where *k*_B_ is the Boltzmann constant, *T* is the temperature, and $\sigma_{\mathrm{APL}}$ is the standard deviation of the APL.

The lateral diffusion constants are estimated by computing the lateral mean-square displacement (MSD $={|\Delta r\left(\tau \right)|}^{2}$ ) of all the lipid phosphate head groups along the membrane plane (*x*,*y* directions). The obtained MSD linear trends are then fitted in a τ range from 1.6 to 14.5 μs to estimate the diffusion constants, *D*, according to Einstein’s equation (MSD =2dDτ , where *d* is the number of dimensions, 2 for lateral diffusivity) and the relative error. We underline that this method does not provide a very accurate estimate of lateral diffusivity, because (i) we are employing a CG model, where the reduced representation of the degrees of freedom of the system implies accelerated dynamics, and (ii) MSD calculations of membrane diffusion are systematically affected by finite size effects.^46^ In any case, we can employ these results as a reliable measure of internal lipid mobility and use them to compare dynamics in the different formulations.

## Results

The main objective of this work is to replicate the structure of extracellular vesicles, with a particular focus on their lipid composition and the resulting properties. Figure 1 provides a schematic overview of the workflow. The process begins with the design and optimization of EV mimic formulations, based on existing literature and simulations. These formulations are then applied to coat a solid, breakable core. The resulting particle library undergoes characterization through both bulk and single-particle techniques, enabling a comparison of their properties to those of natural EVs. The following sections outline each step in greater detail.

**Figure 1:**
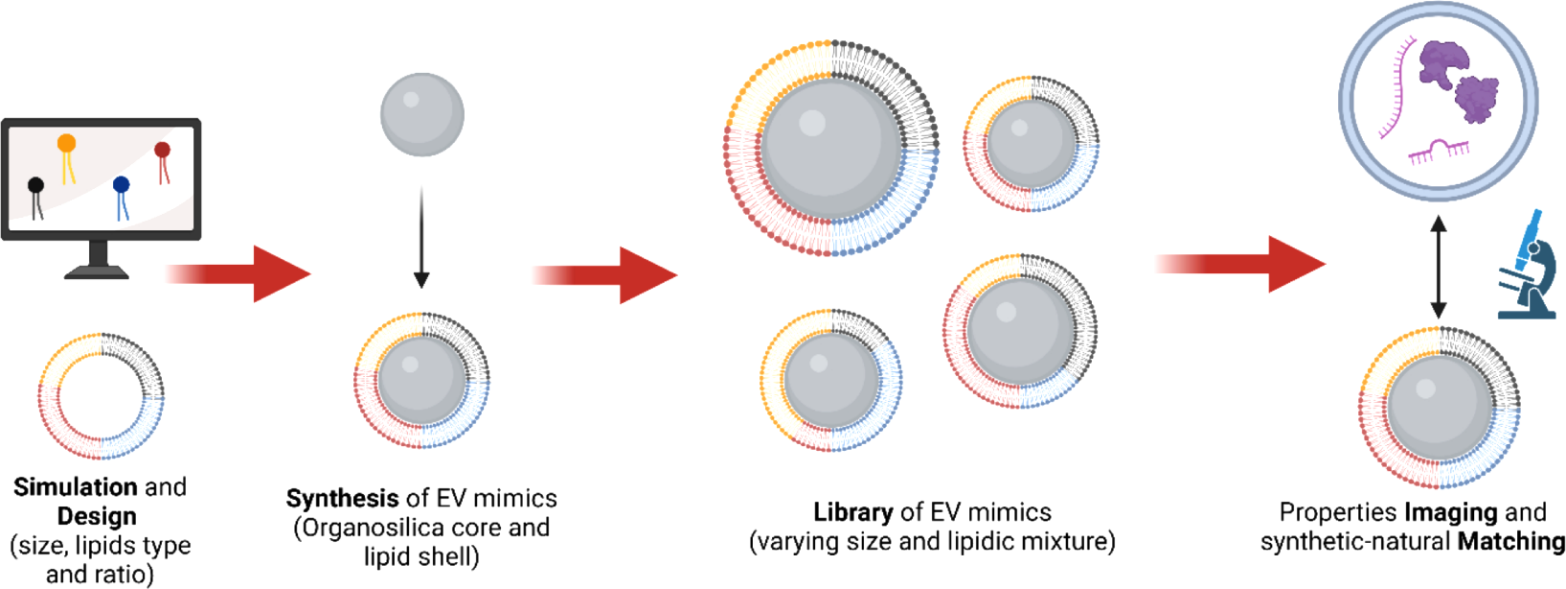
Schematic overview of the workflow presented in this study, from simulation and design to synthesis and characterization.

### Development of EV-Mimicking Lipidic Formulations

The lipid composition plays a crucial role in determining the structure of EVs, as lipids form the characteristic bilayer, and the composition influences the fluidity, rigidity, compartmentalization, and curvature of the membrane. Additionally, the lipid composition affects how EVs are taken up by recipient cells, as certain lipid signatures can influence recognition and subsequent fusion with cell membranes.^47^ PC3-derived EVs (PC3dEVs) were an attractive option here, as their lipid composition has already been identified and published by Ferreri et al.^29^ This natural composition was used as a reference to design several lipid formulations by using commercially available phospholipids and cholesterol. The work by Ferreri et al. analyzed both the mass percentages of the lipid families, reported in Table S1, as well as the fatty acids, reported in Table S2. In our work, only the most abundant species of fatty acids were taken into account, while preserving the same ratio of polar head groups and maintaining the distribution of fatty acid families, i.e., saturated, monounsaturated, and polyunsaturated fatty acids (SFA, MUFA, and PUFA, respectively).

Three lipidic formulations were then developed using a previously optimized formulation (called “3C”), inspired by the COVID-19 BioNTech/Pfizer (BNT162b2) and Moderna (mRNA-1273) vaccines, as a starting point.^30^ The three EV-mimicking formulations were named Mimic 1, Mimic 2, and Mimic 3, and; in this order, they are gradually more complex and more similar to the PC3dEVs. The schematic representations of the lipidic components of the Mimic formulations are presented in terms of lipid classes (Figure 2A) and the content of the different fatty acid species (Figure 2B), in comparison to the PC3dEVs composition.

**Figure 2:**
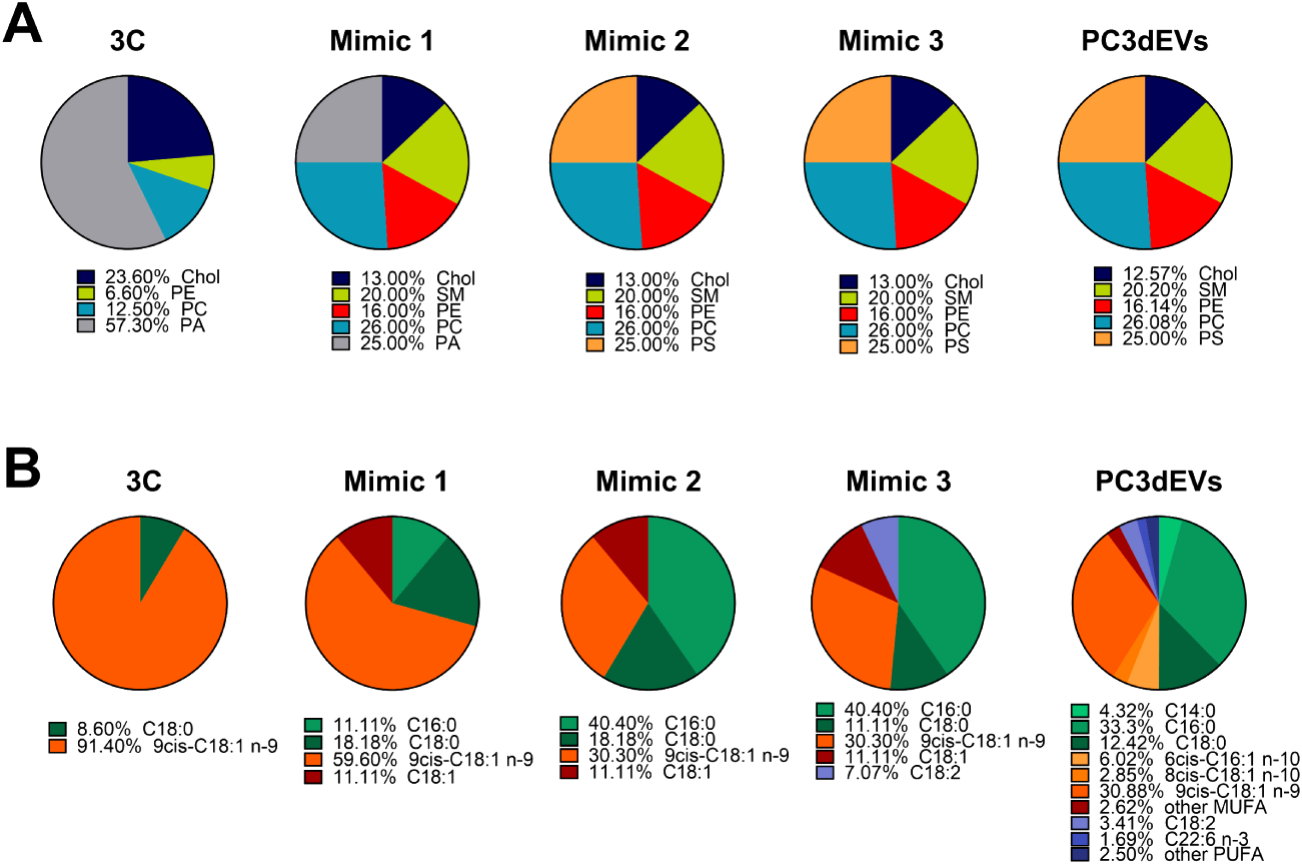
Schematic representation of the mass percentages of A) lipid classes and B) fatty acids of the different designed EV-mimicking formulations: Mimic 1, Mimic 2, and Mimic 3, in comparison to the starting formulation, “3C”, and the simplified natural composition of EVs derived from the PC3 prostate cancer cell line (PC3dEVs) reported by Ferreri et al.^29^

More in detail, the 3C formulation contains an anionic lipid (18:1 phosphatidic acid, PA), which helps in the formation of a homogeneous layer around aminopropyl-functionalized organosilica nanocapsules that show a positive surface charge, guaranteeing an electrostatic interaction between the lipid mixture and the nanoparticles. This formulation was used as a control to evaluate the performance of the EV mimic formulation’s performances.

Mimic 1 is based on a rebalancing of the lipids from the original 3C formulation, with the only addition being sphingomyelins (SM). In this case, the lipidic coating on the nanocapsules still relies on electrostatic interactions as a driving force, mainly caused by the anionic lipid 18:1 PA. On the other hand, in the Mimic 2 formulation, the 18:1 PA was substituted with 16:0 phosphatidylserine (PS). This anionic phospholipid belongs to the only category of lipids that is missing in Mimic 1 with respect to PC3dEVs .^48^ Lastly, the Mimic 3 composition is identical to Mimic 2, except for the substitution of part of the PEGylated lipid (DSPE-PEG) with 18:2 phosphatidylethanolamine (PE), which is done to include PUFAs. Actually, the Mimic 3 formulation includes the most abundant polyunsaturated fatty acid reported for PC3dEVs (with a C18:2 carbon chain), which should enhance the fluidity of the lipidic layer.

As visible in Figure 2, the successive Mimic formulations become gradually more complex and theoretically more similar to the PC3dEVs.

### Assembly and Bulk Characterization of EV Mimics

#### Organosilica Nanocapsules as Core Elements for EV Mimics: Synthesis and Characterizations

The nanocapsules (NCs) employed as the core of the EV Mimics are similar to the NCs previously reported by Prasetyanto and coworkers.^24^ The schematic representation of the synthesis process is depicted in Figure 3A. These NCs contain covalently linked disulfide groups as part of the silica framework. The disulfide bonds can be broken by the reduction of S–S to thiols, which allows for the degradation of the NCs in the presence of a reducing environment (i.e., intracellular cancer levels of glutathione), leading to cargo release.^49^ Furthermore, to facilitate electrostatic interactions with the lipidic shell, the NCs were functionalized with positively charged amine groups (NCs@NH_2_).

**Figure 3:**
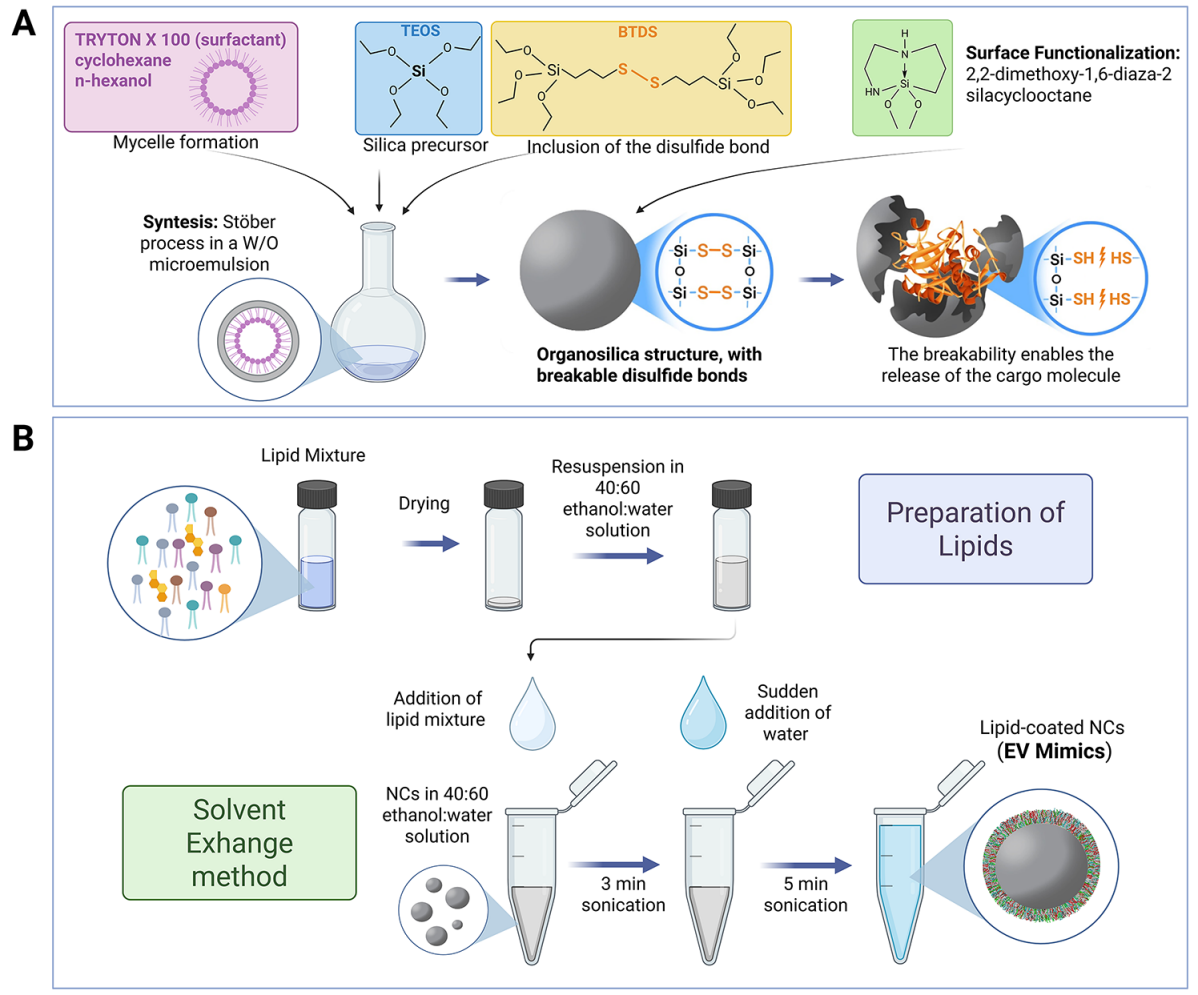
A) Schematic representation of the synthesis of organosilica nanocapsules procedure with the main reagents employed. B) Schematic representation of the procedure followed to coat the organosilica nanocapsules, which involves the solvent exchange method.

Transmission electron microscopy (TEM) imaging reveals the presence of spherical NCs with a diameter of about 70 nm (Figure S2A). Dynamic light scattering (DLS) measurements of the pristine NCs and the functionalized NCs@NH_2_ are in agreement with TEM images, exhibiting a hydrodynamic diameter peak at 70 nm (polydispersity index, PdI equal to 0.10) and 80 nm (with PdI of 0.21), respectively (Figure S3A) with homogeneous and monodispersed size distributions. The zeta potential of the NCs (Figure S3B) changed from the typical negative charge of silica materials (−19.7 ± 2.4 mV) to a positive charge (+23 ± 5.6 mV) due to the amine functionalization of the NCs@NH_2_. This shift in charge, together with the increase in hydrodynamic diameter, demonstrates that the positively charged functional moieties were successfully anchored to the NC surface. Further characterization of the NCs@NH_2_ is presented in Figure S3.

#### Mimic Formulations Successfully Coat Organosilica NCs, Resulting in EV Mimics

To create a nanoconstruct with an EV-biomimetic surface for interaction with the biological environment, the various Mimic formulations were utilized to coat NC@NH_2_ using the solvent exchange method,^30^ which drives the self-assembly of lipids around the nanoparticles. The obtained lipid-coated NC@NH_2_ (EV Mimics) were characterized by means of DLS and Zeta potential analysis, showing a general shift from positive (+27.1 ± 6.22 mV) values of the NCs@NH_2_ to negative ones for the NCs@NH_2_ coated with the different lipidic formulations, as reported in Table 3 and Figure 4A. Furthermore, an increase in the nanoparticle’s hydrodynamic radius was observed (Figure 4B), suggesting that the NC@NH_2_ were effectively coated.

**Figure 4:**
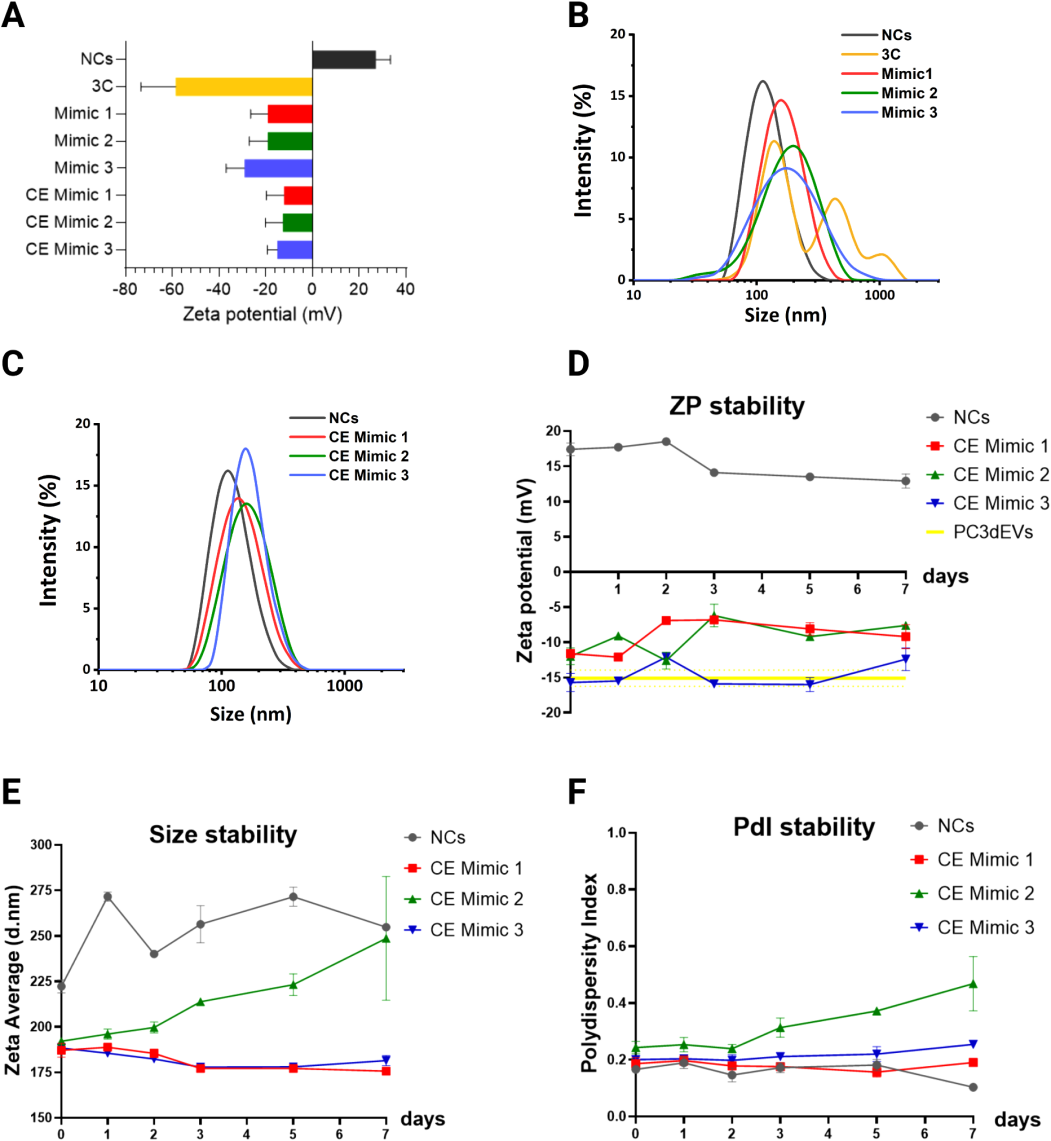
EV Mimics characterization. A) Zeta potential measurements; B) DLS measurements of uncoated NCs and NCs coated with formulations 3C, Mimic 1, Mimic 2, and Mimic 3; C) DLS measurements of NCs coated with the cholesterol-enhanced mimic formulations. D) Stability evaluation of the Zeta potential, E) average size, and F) PdI of NCs and EV Mimics.

**Table 3:** Summary of Size and Zeta Potential Measurements of the EV Mimics

	Size		
EV Mimics	*Z*-Average (nm)	PdI	Zeta potential (mV)
3C	448.6	0.75	-58.6 ± 14.8
Mimic 1	162.1	0.26	-19.1 ± 7.3
Mimic 2	174.5	0.36	-19.2 ± 7.9
Mimic 3	166.8	0.37	-29.1 ± 7.9
CE Mimic 1	187.1	0.19	-12.2 ± 7.55
CE Mimic 2	192.1	0.24	-12.7 ± 7.46
CE Mimic 3	188.3	0.20	-15.1 ± 4.25

However, the 3C formulation produced a size distribution with multiple peaks, indicating the formation of aggregates or multilayered lipid coatings around the NCs. This is supported by the Zeta potential values, reaching highly negative values (−58.6 ± 14.8 mV), which can denote the presence of a high number of lipids surrounding the NCs. The Mimic formulations, particularly Mimic 1, showed more regular size distributions, with an average hydrodynamic diameter between 150 and 200 nm and a Zeta potential ranging approximately from −10 to −30 mV. Mimic 2 and Mimic 3 formulations produced a wider size distribution than Mimic 1.

Cholesterol is known to enhance the stability of a lipid bilayer and alter its rigidity.^50,51^ Moreover, according to some research works, the content of cholesterol in prostate cancer-derived EVs may be higher than the mass percentage of 13% used in the initial mimic formulations.^52,53^ In line with these studies, cholesterol-enhanced mimic formulations (CE Mimics), with a higher mass percentage of cholesterol—30% (Figure S1)—were produced and tested, obtaining more homogeneous size distributions, as evidenced by Figure 4C. Table 3 summarizes the size and Zeta potential results collected for EV Mimics.

To corroborate the quality of the cholesterol-enhanced lipid formulations, stability tests were performed. Both uncoated NCs and EV Mimics were monitored through DLS for 1 week; the resulting values of Zeta potential, average size, and polydispersity index are respectively reported in Figure 4D–F. With regard to the size, we observed that NCs quickly tend to aggregate, while EV Mimics remained more stable over time. In particular, the average size and PdI values of CE Mimic 1 and CE Mimic 3 formulations remained constant throughout the whole period of observation. On the other hand, an increase in size was registered for CE Mimic 2. As for the Zeta potential, CE Mimic 1 and CE Mimic 2 suffered from a slight decrease in the absolute value over time. Notably, CE Mimic 3 instead showed a constant Zeta potential value, which also closely overlaps with the one registered for reference natural EVs.

#### Computational Modeling Guides the Optimization of Lipid/NCs Ratio

To further improve the nanoparticle coating process, the optimal amount of lipids required for the formation of a single bilayer on their surface was estimated. For this purpose, experimental work and computational estimates were combined to evaluate the surface area exposed by the NC@NH_2_, as well as the number of lipid molecules necessary to optimally coat the said surface area.

The diameter and the wall thickness of NC@NH_2_ were estimated from TEM images (exemplified in Figure S2A), obtaining an average diameter of 79.4 ± 11.6 nm. Indeed, as reported in Figure S2B, more than 70% of NC@NH_2_ have a diameter between 70 and 90 nm and an estimated wall thickness of 3.68 ± 0.89 nm (Figure S2C). The weight depends on the atomic structure of silica, and the nanoparticle’s density is different from that of the bulk material (crystalline silica).^54^ Literature reports different densities for silica nanoparticles, ranging from 1.4 to 2.6 g/cm^3^.^31,54–56^ If a silica density of 1.87 g/cm^3^ is assumed, as reported by Kimoto and colleagues^31^ for silica nanoparticles synthesized with a sol–gel process, similar to the one employed in this work, the estimated weight of a single NC is 1.3 × 10^–6^ g (Table S3).

The computational modeling of a spherical lipid bilayer structure (see Experimental Section) provides an estimate of the number of molecules in its upper and lower leaflets. Under the assumption that nanocapsules are perfect spheres, this calculation provided a guideline on the amount of each lipid species required to form an optimal, single bilayer coating on an organosilica sphere of a given diameter. The mass of a single bilayer shell for a range of NCs’ diameters and for each of the Mimic formulations was then calculated. The obtained values are reported in Table S4. This gave an optimal theoretical mass ratio of NCs to lipids between 1.4 and 2.6, as reported in Table S5 . Successively, the NCs’ masses were weighed according to their size distribution, as documented in Table S6. The results of such calculations indicate an optimal range of NCs/lipids ratios between 1.91 and 2.30.

With the aim to verify if the data obtained through the theoretical approach were close to real conditions, different mass ratios of NC@NH_2_ and lipid formulations (namely, 1:1, 2:1, 3:1, 4:1, 5:1, and 10:1 NC@NH_2_/lipids) were experimentally tested, employing the most promising formulations (CE Mimic 1 and CE Mimic 3). The quality of the coating of NC@NH_2_ with the different ratios was then evaluated and compared using DLS. More specifically, to determine the optimal ratio, we took advantage of the tendency of NCs to naturally aggregate in PBS (as shown in Figure S4), whereas properly coated nanoparticles are expected to maintain their size. Through DLS measurements, we indeed observed an increase in size and polydispersity index by reducing the amount of lipids employed.

NCs coated with a 2:1 NCs/lipids mass ratio, as visible from Figure 5A,B, showed the smallest average size and the lowest PdI. The 1:1 ratio also resulted in a reasonably good size distribution; however, it was slightly less uniform compared to the 2:1 ratio, possibly due to the presence of an excess of lipids. On the other hand, 3:1, 4:1, 5:1, and 10:1 NCs/lipids mass ratios showed multiple peaks at much higher values and higher polydispersity indexes. This was attributed to aggregation occurring when NCs are not fully coated by lipids. Remarkably, 2:1 was confirmed as the best ratio from the experimental point of view. Therefore, the experimental evidence matches the estimated best NCs/lipids optimal range.

**Figure 5:**
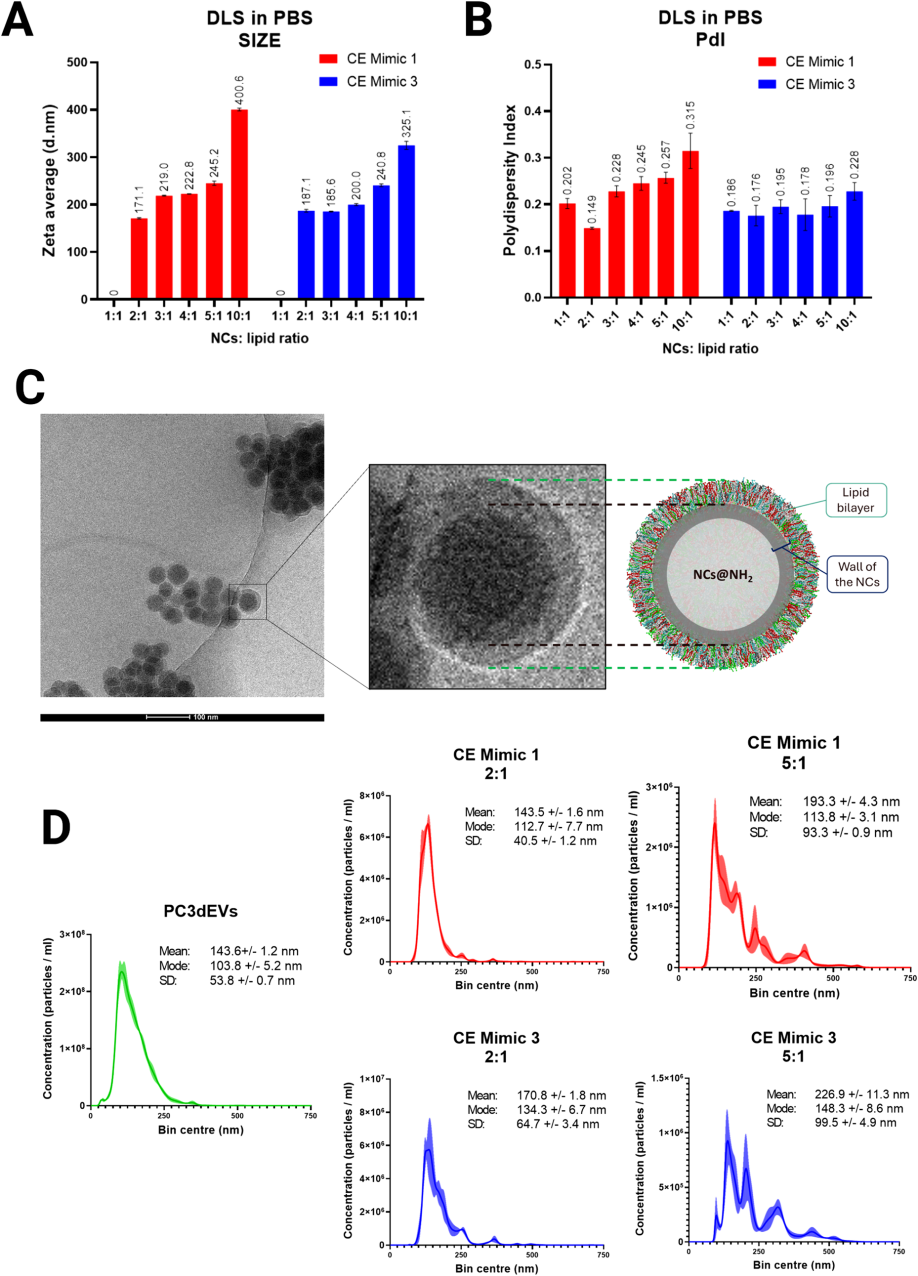
Characterizations for the optimization of the NCs/lipids mass ratio: A) zeta average values and B) polydispersity index values resulting from DLS measurements of NCs coated CE Mimic 1 (red) or CE Mimic 3 (blue) formulations. Measurements were performed at different NCs/lipids mass ratios in PBS, since uncoated NCs tend to aggregate in PBS, while proper coating prevents NCs’ aggregation. C) Cryo-TEM image of an EV Mimic (CE Mimic 1), supplemented with a close-up view of the Cryo-TEM image, matched with the theoretical representation of EV Mimics, assumed in the computational model. D) Illustrative results of Nanoparticle Tracking Analysis (NTA) of natural PC3-derived EVs (green), and NCs coated with CE Mimic 1 (red) or CE Mimic 3 (blue) formulations at NCs/lipids mass ratios equal to 2:1 or 5:1.

By employing the 2:1 ratio, the obtained EV Mimics appear as single nanoparticles coated with an organic shell attributed to a lipid layer, as observable from cryo-transmission electron microscopy (Cryo-TEM) images reported in Figure 5C. In this way, the nanoconstruct’s appearance is in agreement with the theoretical one assumed for computational modeling of EV Mimics. In Figure S5, further Cryo-TEM images are displayed, showing the differences between the different lipid-coated and uncoated NCs@NH_2_. The EDS analysis confirmed the presence of Si atoms in the internal core of the EV Mimics (Figure S6).

Moreover, DLS data were confirmed by Nano Tracking Analysis (NTA), as exemplified in Figure 4D. The complete set of NTA measurements is instead reported in Figures S7 and S8. Remarkably, the assembly of EV Mimics with the 2:1 ratio resulted in nanoparticles with a size distribution very close to that of natural PC3dEVs.

### Molecular Simulations Characterize Lipid Dynamics at the Nanoscale

To gain a further understanding of the EV-Mimicking formulations, we performed molecular dynamics (MD) simulations of only the three CE Mimic formulations, in view of their superior behavior shown above. To this end, molecular models were created using the coarse-grained (CG) modeling scheme MARTINI 3.0, in which groups of 3–4 heavy atoms are represented by single particles interacting among each other according to properly parameterized potentials.^37,57^ Using such a lower modeling resolution, with respect to atomistic models, allows for reaching the space and time scales relevant to the study of lipid membrane dynamics, while also preserving important details of the intermolecular interactions.

Models of CE Mimic 1, 2, and 3 were developed, and nanometer-sized portions of single-stack lipid bilayer membranes (see schemes in Figure 6A) were simulated, focusing our MD study on two configurations: (i) free/unsupported membrane, with the bilayer simply immersed in water, and (ii) supported membrane, where the lower leaflet of the bilayer is noncovalently bound to a porous organosilica surface functionalized with diaza-silane (see also the Experimental Section).

**Figure 6:**
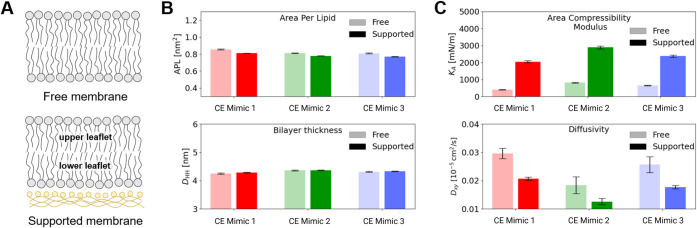
Characterization of the CE Mimic formulations from MD simulations. A) Simulated configurations: free/unsupported membrane (top) and grafted/supported membrane (bottom). B) Membrane structure at equilibrium (*T* = 310 K): area per lipid (top) and bilayer thickness $D_{\mathrm{HH}}$ (bottom), both reported as averages. C) Membrane dynamics at equilibrium (*T* = 310 K): area compressibility modulus, $K_{A}$ (top); and average lateral diffusivity quantified for lipid heads in the upper leaflet (bottom).

The simulations of free/unsupported bilayer membranes are representative of liposomes as well as portions of the EV Mimic envelope that would be locally detached from the porous organosilica surface (as a water layer can form between the hard-shell NP and the coating envelope). As explained in detail in the Experimental Section, MD simulations allowed us to characterize the equilibrium behavior of the different EV Mimic formulations under standard conditions of temperature and pressure (*T* = 310 K and *p* = 1 atm).

We first evaluated how the structural features of the membranes depend on their composition and their binding to the organosilica surface. The area per lipid (APL) and bilayer thickness are reported in Figure 6B. We observed that differences between CE Mimic formulations do not trigger large variations in the structural parameters, as also supported by the radial distribution functions (Figure S9). We then moved on to the characterization of the internal dynamics of EV mimic envelopes, as this is a key aspect for their envisaged applications. To measure the dynamics of the envelopes, we computed the area compressibility modulus *KA*, which quantifies the resistance of the bilayer to lateral compression (i.e., along the *xy* axes), and the average lateral diffusivity of the lipids (again along the *xy* axes, computed across the upper leaflet; see Figure 6A), which quantifies lipid mobility along the plane of the bilayer. The lateral diffusivity is computed by evaluating the MSD of the phosphate groups of all lipid heads (see Experimental Section).

The results are reported in Figure 6C, showing that dynamics are significantly reduced when binding to the organosilica surface occurs (in terms of both area compressibility modulus and lateral diffusivity of the lipids). The binding onto the surface of the NCs implies reduced lipid mobility also in the upper leaflet. Moreover, we notice that dynamics vary significantly between the different Mimic formulations, which indicates that CE Mimic 1 has both the lowest *K*_*A*_ and the highest lateral diffusivity, followed by CE Mimic 3 and CE Mimic 2.

The MD analysis, therefore, provides further details on the molecular-scale behavior of the different envelope formulations. The results show substantial similarity in structural properties (Figure 6B), but striking differences are observed in the inner dynamics of the envelopes for different lipid compositions, showing in particular, how the CE Mimic 2 envelope has lower dynamicity than the other two formulations (Figure 6C). Comparing the formulations CE Mimic 2 and CE Mimic 3, which are equal in terms of lipid head type composition (Figure 1A), we can hypothesize that differences in terms of lipid tail type composition (Figure 1B) are instead at the basis of such variable dynamics. Accordingly, it appears that the higher proportion of saturated lipid tails in formulation CE Mimic 2 contributes to the greater stiffness of the envelope. Instead, CE Mimic 1, although diverging from CE Mimic 3 in both lipid head and tail type composition, shows dynamics similar to those of CE Mimic 3 (Figure 6). As a whole, MD unveils further physical details about the properties of the systems, in particular, on the internal dynamics of the lipid bilayers, which are hardly accessible via experiments. When coupled with the experimental results presented herein, MD modeling indicates that higher compressibility and diffusivity of the lipidic membrane correlate with better quality in terms of nanocarrier properties (see also the following sections).

### Single-Particle Analysis of EVs and EV Mimics

To further enhance our understanding of the characteristics of the proposed EV Mimics, we complemented bulk characterization techniques with single-particle analysis methods. While bulk characterizations provide valuable insights into overall properties, such as average size distribution, surface charge, and composition, they can mask the heterogeneity within a population.

### High-Resolution Flow Cytometry Reveals Differences in Lipid Coating Efficiency of Mimic Formulations

To quantify the coating efficiency of the different lipid formulations, we employed high-resolution flow cytometry, which allows for the multiparametric analysis of individual particles in a high-throughput manner. NCs were labeled with Atto488 dye, and the lipid staining was performed with the lipophilic dye DiD. The data presented in Figure 7A display the gating and representative dot plots. Figure 7B shows the percentage of double-positive events, which indicates the degree of lipid coating obtained for the different EV Mimics. The complete set of dot plots is presented in Figure S10.

**Figure 7:**
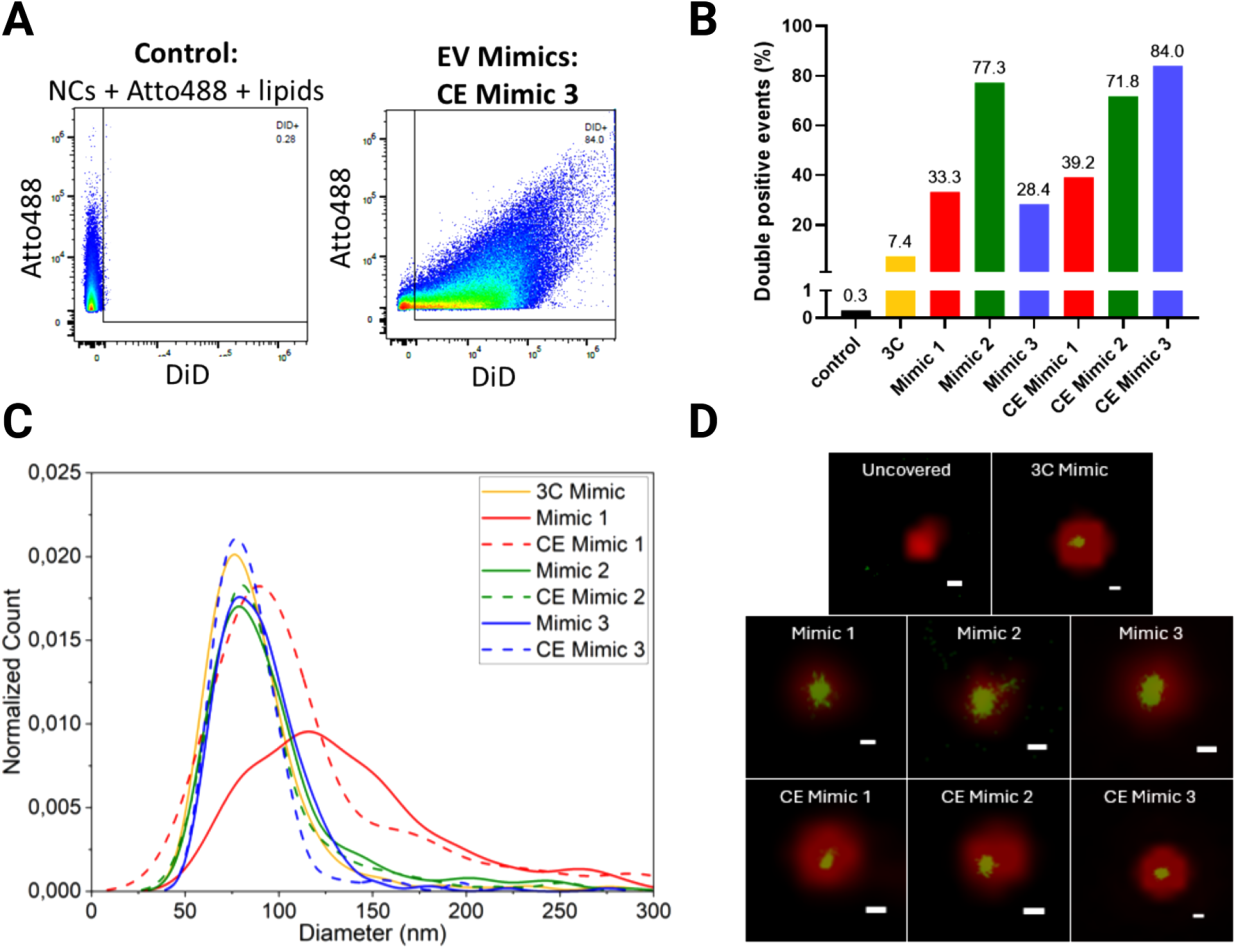
A) High-resolution flow cytometry analysis of EV Mimics: illustrative dot plots of control samples and EV Mimics (CE Mimic 3). Each dot represents a single nanoparticle. In EV Mimic samples, NCs were labeled with Atto488 (*Y*-axis) and lipids were stained with the lipophilic dye DiD (*X*-axis). The detection threshold was set above unstained NCs, ensuring that each event is a stained NC. B) Percentages of double-positive events in high-resolution flow cytometry analysis of the EV Mimics. C) Size distribution of the EV Mimics as determined by clustering of Nile Red single-molecule localizations. D) Examples of colocalized images of diffraction-limited signal from Atto647-labeled silica cores (red) and super-resolved single-molecule Nile Red localizations (green). Scale bars represent 100 nm.

The results show a significant difference in the percentages of double-positive events among the various EV Mimics, which clearly outperformed the 3C formulation. Mimic 2 shows a distinctly higher percentage of double-positive events than Mimic 1 and Mimic 3. It was also observed that the cholesterol-enhanced variants show a generally higher degree of double-positive events, particularly for CE Mimic 3, which shows the best performance of all formulations, confirming the role of cholesterol in stabilizing the double layer.^50,51^ These results suggest that small changes in the lipid formulation can affect the efficiency of NC coating, which, in turn, can have significant implications for eventual functionality.

### Single-Molecule Localization Microscopy Using a Solvatochromic Dye Enables Polarity Probing at High Resolution

SMLM was also used to probe the lipid bilayer. The organosilica cores were stained with Atto647, while Nile Red was employed to probe the lipids. Nile Red binds transiently to the hydrophobic regions of the lipid bilayer, emitting a detectable fluorescence signal upon binding. To prevent cross-bleeding of the Nile Red signal, NCs and lipids were imaged sequentially and then aligned.

First, we confirmed that uncovered particles without a lipid bilayer show no interaction with the Nile Red probe, while it showed binding events on the lipid-coated nanoparticles (see Figure 7D). Additionally, clustering the Nile Red signal gives further indication of the size range, as reported in Figure 7C. This information is important, as it is possible for aggregates of multiple silica particles to be enclosed within a bilayer. We observe a relatively broad size range from roughly 50–150 nm for nearly all EV Mimics, with a few larger aggregates. In particular, the Mimic 1 formulations show a significant population of larger sizes.

Nile Red also has another key feature, which is that its emission wavelength is highly sensitive to its local environment, with lower polarity regions causing a blue shift and more polar regions inducing a red shift. We took advantage of this feature to compare the membrane polarities of both the natural EVs and EV Mimics, obtaining valuable insight into their structural physicochemical similarities.

By placing a blazed diffraction grating before the detector, the emitted signal of single Nile Red molecules is separated into two paths: the spatial domain, or the position of the molecule, and the spectral domain, which shows the emission spectrum (as exemplified in Figure 8A and schematized in Figure 8B). The spatial domain is emitted as a typical point spread function (PSF), which can be fitted to obtain the position in *x* and *y* at that specific time point. The spectral domain can be fitted to obtain the peak wavelength of the emission. This was done over thousands of frames to gather significant statistics at the single-molecule level for each particle.

**Figure 8:**
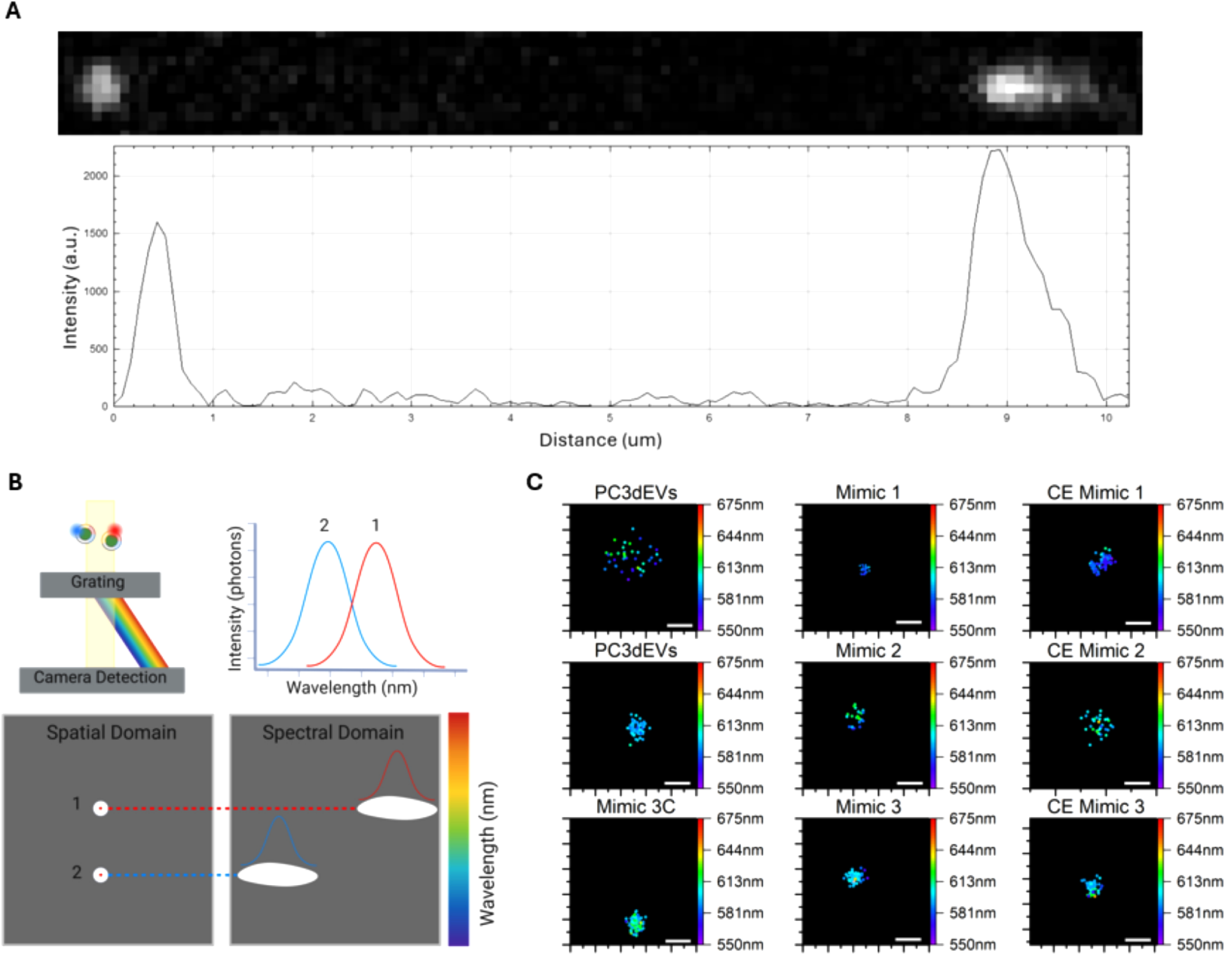
A) Example cropped image of a localization and the corresponding spectral footprint of a Nile Red binding event. The intensity profile versus distance is plotted below it. These distributions are fitted to obtain the spatial coordinates and the peak wavelength. B) Schematic representation of the spatial and spectral detection using a blazed diffraction grating and solvatochromic dye. C) Representative single-molecule reconstructed images of EV and EV Mimic particles, colored by the wavelength (in nm) of the localization emission. Scale bars represent 100 nm.

Figure 8C shows example clusters for all EV Mimics as well as the PC3dEVs themselves. The *XY* coordinates of each Nile Red localization are combined across all frames to produce reconstructed spatial maps of the binding events, representing the lipid-covered particles at nanoscale precision. Example spectral distributions for single particles are shown in Figure S11.

To gain a comprehensive understanding of the entire population, we analyzed all localizations associated with validated particles and plotted the distribution of their peak wavelengths (Figure 9). This result shows which EV Mimics are most similar to PC3dEVs across the entire population. A cross-correlation analysis was conducted to assess the similarity between the distributions. Notably, CE Mimic 3 (i.e., with the highest amount of cholesterol) and both variants of Mimic 1 (at low and high levels of cholesterol) exceeded the threshold of 0.9, indicating a good match with the polarity of PC3dEVs. Thus, despite the simplifications introduced with respect to PC3dEVs, we were able to reproduce some of the natural EV’s physicochemical properties. The higher degree of similarity between CE Mimic 1 and CE Mimic 3 compared to CE Mimic 2 also correlates well with the earlier explored lipid dynamics (Figure 6).

**Figure 9:**
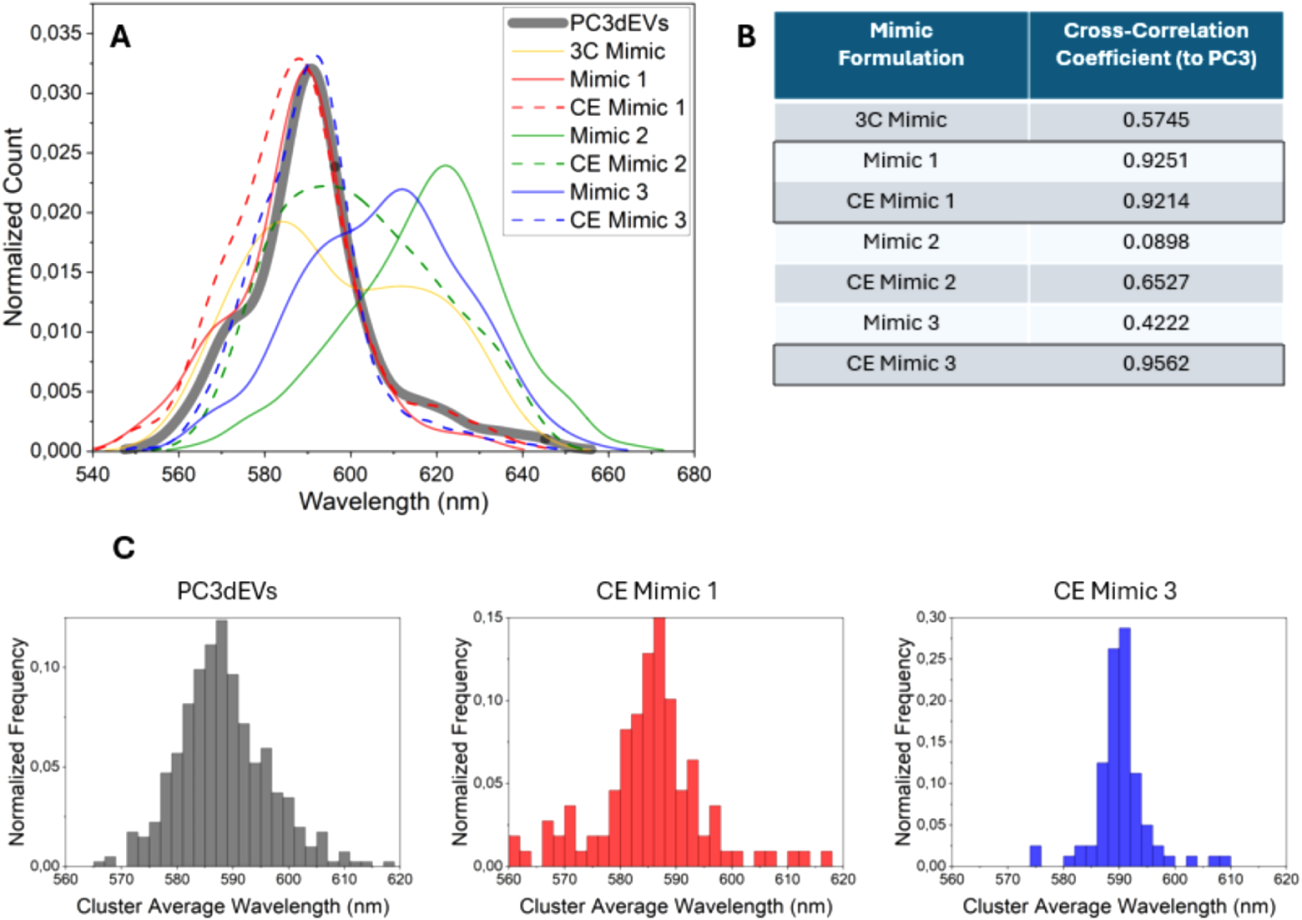
A) Distribution of all localization wavelengths for validated clusters of EVs and EV Mimics, visualized and B) with calculated cross-correlation coefficient for EV Mimics compared to the PC3dEVs. Values over 0.9 are considered to be significantly similar. C) Histograms displaying the cluster-average wavelengths, where for each validated cluster all localization peak wavelengths are averaged and taken as a single data point for PC3dEVs and all EV Mimics. This shows the heterogeneity among particles within the same population.

A crucial factor to consider is the heterogeneity of the population. Not all PC3dEVs and EV Mimics exhibit the same polarity, and it is highly probable that the EVs of interest belong to a specific subpopulation. Therefore, it is essential to study the population’s heterogeneity. To this end, we examined the cluster average wavelength, defined as the mean peak wavelength of all localizations associated with a single particle (Figure 9C). This approach provides insights into interparticle heterogeneity that cannot be obtained through bulk characterization techniques. The PC3dEV population demonstrates slightly greater heterogeneity in lipid polarity than that of the most similar EV Mimics, which is not unexpected. Certain natural EV populations can overlap in size despite originating from distinct pathways. Since PC3dEVs were obtained through differential fractionation, it is likely that the sample contains various subpopulations. With regard to the EV Mimics, CE Mimic 1 appears significantly more heterogeneous than CE Mimic 3. This could be a result of the formulation itself, but perhaps also the coverage, as Mimic 1 formulations showed more aggregation (Figure 7C), which could lead to more unusual structures and lipid interfaces.

Differences are also observed in the intraparticle heterogeneity (Figure S12), which shows a great deal of variance in the PC3dEV population, with some EVs being very homogeneous and others very heterogeneous. The EV Mimics show a narrower distribution, with CE Mimic 3 again being slightly more homogeneous than CE Mimic 1.

With regard to mimicking the PC3dEVs, the more homogeneous nature of the EV Mimics could mean that they do not mimic the entire population, which is a factor to consider. However, it is a clear benefit to mimic a specific subset of the EV population in the future. Taking this into consideration, together with the differences observed in polarity, as well as the coating efficiency and the MD analysis, the cholesterol-enhanced version of Mimic 3 seems to be the most suitable candidate as an EV-based nanocarrier.

## Conclusions

We developed a method to formulate, prepare, and characterize biomimetic nanoparticles inspired by natural extracellular vesicles (EVs). Our EV Mimics were planned and prepared as core–shell hybrid structures, consisting of a degradable organosilica nanocapsule for efficient cargo delivery and controlled release, surrounded by a biomimetic lipid shell that emulates the lipid composition of natural EVs. Three different lipidic mixtures were designed based on lipidomic data of PC3-derived EVs, accounting for the mass percentages of the lipid families and the fatty acids. These EV-inspired formulations were shown to successfully coat the NCs, with cholesterol-enhanced EV Mimics displaying more homogeneous size distributions.

To optimize the lipid bilayer coating of the organosilica core, we employed predictive modeling, which was further validated experimentally. Supramolecular simulations also showed differences in the lipid dynamics for the various Mimic formulations. The size distribution and Zeta potential were analyzed among the different EV-mimicking formulations and compared to the natural EVs, showing a strong degree of similarity between them. In addition to these conventional characterization methods, high-resolution flow cytometry was employed to quantify the lipid coating efficiency, and a Nile Red-based single-molecule localization microscopy technique was used to specifically probe and map the polarity of the individual EV Mimics and compare it to natural EVs, highlighting differences in the membrane environment across formulations. From this multiparametric analysis, a single Mimic formulation emerged as the most promising candidate. This is also the formulation most similar to the natural composition of PC3dEVs, with an additional enhancement in the cholesterol content.

Together, these results establish a strategy for the rational design of nature-inspired lipid-coated nanoparticles. This approach allows for easy, scalable, and cost-effective production of artificial lipid particles with tunable properties inspired by natural EVs. Importantly, the use of polarity-sensitive single-molecule imaging offers a functional readout of the nanoscale membrane environment relevant to biological interaction. Finally, we developed supramolecular models that offer insights into the deposition and dynamics of lipids in the bilayer—information that is challenging to access experimentally. Overall, this work provides a guideline for developing fully synthetic and biomimicking nanocarriers that emulate key physical traits of natural EVs: nature’s own, highly efficient delivery system.

## Supplementary Material



## Notes

This material is available free of charge at https://pubs.acs.org/doi/. The Supporting Information is available free of charge at https://pubs.acs.org/doi/10.1021/acsanm.5c01459.

Additional experimental details, materials, and methods, including experimental and simulation data: mass percentage of different lipid species present in PC3-derived EVs and in the mimic formulations; nanocapsules synthesis and characterization; details on optimization of lipid/NCs ratio; nanoparticle tracking analysis; additional cryo-TEM images and EDS analysis; additional simulations computed for all lipids; high-resolution single-particle analysis; cell internalization tests of NCs and EV Mimics (PDF)

## ◆

Instituto de Nanociencia y Materiales de Aragón (INMA), CSIC-Universidad de Zaragoza, Campus Rio Ebro, Edificio I + D + I, C/Poeta Mariano Esquillor, s/n, 50018, Zaragoza, Spain; Department of Chemical and Environmental Engineering, Institute of Nanoscience and Materials of Aragon, Universidad de Zaragoza, Zaragoza, Spain; Instituto de Investigación Sanitaria de Aragón (IIS Aragón), Avda. San Juan Bosco, 13, 50009, Zaragoza, Spain; Networking Research Center in Biomaterials, Bioengineering and Nanomedicine (CIBERBBN), Instituto de Salud Carlos III, 28029, Madrid, Spain

## References

[1] Gote V., Bolla P. K., Kommineni N., Butreddy A., Nukala P. K., Palakurthi S. S., Khan W. (2023). A Comprehensive Review of mRNA Vaccines. Int. J. Mol. Sci..

[2] Modena M. M., Rühle B., Burg T. P., Wuttke S. (2019). Nanoparticle Characterization:
What to Measure?. Adv. Mater..

[3] Khan K. U., Minhas M. U., Badshah S. F., Suhail M., Ahmad A., Ijaz S. (2022). Overview of Nanoparticulate
Strategies for Solubility Enhancement
of Poorly Soluble Drugs. Life Sci..

[4] Puri S., Mazza M., Roy G., England R. M., Zhou L., Nourian S., Anand Subramony J. (2023). Evolution
of Nanomedicine Formulations
for Targeted Delivery and Controlled Release. Adv. Drug Delivery Rev..

[5] Rommasi F., Esfandiari N. (2021). Liposomal Nanomedicine: Applications
for Drug Delivery
in Cancer Therapy. Nanoscale Res. Lett..

[6] Halwani A. A. (2022). Development
of Pharmaceutical Nanomedicines: From the Bench to the Market. Pharmaceutics.

[7] Anselmo A. C., Mitragotri S. (2019). Nanoparticles
in the Clinic: An Update. Bioeng. Transl. Med..

[8] Nyström A. M., Fadeel B. (2012). Safety Assessment
of Nanomaterials: Implications for
Nanomedicine. J. Controlled Release..

[9] Tenchov R., Sasso J. M., Wang X., Liaw W. S., Chen C. A., Zhou Q. A. (2022). Exosomes Nature’s Lipid Nanoparticles, a Rising
Star in Drug Delivery and Diagnostics. ACS Nano.

[10] Elsharkasy O. M., Nordin J. Z., Hagey D. W., de Jong O. G., Schiffelers R. M., Andaloussi S. E., Vader P. (2020). Extracellular Vesicles as Drug Delivery
Systems: Why and How?. Adv. Drug Delivery Rev..

[11] Kalluri R., LeBleu V. S. (2020). The Biology, Function,
and Biomedical Applications
of Exosomes. Science.

[12] Gurung S., Perocheau D., Touramanidou L., Baruteau J. (2021). The Exosome Journey:
From Biogenesis to Uptake and Intracellular Signalling. Cell Commun. Signaling..

[13] Bahrami A., Moradi Binabaj M., Ferns G.A. (2021). Exosomes: Emerging Modulators of
Signal Transduction in Colorectal Cancer from Molecular Understanding
to Clinical Application. Biomed. Pharmacother..

[14] Urbanelli L., Magini A., Buratta S., Brozzi A., Sagini K., Polchi A., Tancini B., Emiliani C. (2013). Signaling
Pathways
in Exosomes Biogenesis, Secretion and Fate. Genes.

[15] Banks W. A., Sharma P., Bullock K. M., Hansen K. M., Ludwig N., Whiteside T. L. (2020). Transport of Extracellular Vesicles across the Blood-Brain
Barrier: Brain Pharmacokinetics and Effects of Inflammation. Int. J. Mol. Sci..

[16] Abdelsalam M., Ahmed M., Osaid Z., Hamoudi R., Harati R. (2023). Insights into
Exosome Transport through the Blood–Brain Barrier and the Potential
Therapeutical Applications in Brain Diseases. Pharmaceuticals.

[17] Tan F., Li X., Wang Z., Li J., Shahzad K., Zheng J. (2024). Clinical Applications
of Stem Cell-Derived Exosomes. Signal Transduct.
Target. Ther..

[18] Muthu S., Bapat A., Jain R., Jeyaraman N., Jeyaraman M. (2021). Exosomal Therapy—a New Frontier in Regenerative
Medicine. Stem Cell Investig.

[19] Kim H. I., Park J., Zhu Y., Wang X., Han Y., Zhang D. (2024). Recent Advances in Extracellular Vesicles for Therapeutic Cargo Delivery. Exp. Mol. Med..

[20] Rosso G., Cauda V. (2023). Biomimicking Extracellular
Vesicles with Fully Artificial Ones: A
Rational Design of EV-BIOMIMETICS toward Effective Theranostic Tools
in Nanomedicine. ACS Biomater. Sci. Eng..

[21] Sakai-Kato K., Yoshida K., Takechi-Haraya Y., Izutsu K. I. (2020). Physicochemical
Characterization of Liposomes That Mimic the Lipid Composition of
Exosomes for Effective Intracellular Trafficking. Langmuir.

[22] Lu M., Huang Y. (2020). Bioinspired
Exosome-like Therapeutics and Delivery Nanoplatforms. Biomaterials.

[23] Evers M. J. W., van de Wakker S. I., de Groot E. M., de Jong O. G., Gitz-François J. J. J., Seinen C. S., Sluijter J. P. G., Schiffelers R. M., Vader P. (2022). Functional siRNA Delivery
by Extracellular Vesicle–Liposome Hybrid Nanoparticles. Adv. Healthcare Mater..

[24] Prasetyanto E. A., Bertucci A., Septiadi D., Corradini R., Castro-Hartmann P., De Cola L. (2016). Breakable Hybrid Organosilica Nanocapsules
for Protein Delivery. Angew. Chem., Int. Ed..

[25] Sancho-Albero M., Rosso G., De Cola L., Cauda V. (2023). Cargo-Loaded Lipid-Shielded
Breakable Organosilica Nanocages for Enhanced Drug Delivery. Nanoscale.

[26] Probert C., Dottorini T., Speakman A., Hunt S., Nafee T., Fazeli A., Wood S., Brown J. E., James V. (2019). Communication
of Prostate Cancer Cells with Bone Cells via Extracellular Vesicle
RNA; a Potential Mechanism of Metastasis. Oncogene.

[27] Cavallaro S., Pevere F., Stridfeldt F., Görgens A., Paba C., Sahu S. S., Mamand D. R., Gupta D., El Andaloussi S., Linnros J. (2021). Multiparametric Profiling
of Single Nanoscale Extracellular Vesicles by Combined Atomic Force
and Fluorescence Microscopy: Correlation and Heterogeneity in Their
Molecular and Biophysical Features. Small.

[28] Maas S. L. N., Breakefield X. O., Weaver A. M. (2017). Extracellular Vesicles:
Unique Intercellular Delivery Vehicles. Trends
Cell Biol..

[29] Ferreri C., Sansone A., Buratta S., Urbanelli L., Costanzi E., Emiliani C., Chatgilialoglu C. (2020). The N-10 Fatty
Acids Family in the Lipidome of Human Prostatic Adenocarcinoma Cell
Membranes and Extracellular Vesicles. Cancers.

[30] Conte M., Carofiglio M., Rosso G., Cauda V. (2023). Lipidic Formulations
Inspired by COVID Vaccines as Smart Coatings to Enhance Nanoparticle-Based
Cancer Therapy. Nanomaterials.

[31] Kimoto S., Dick W. D., Hunt B., Szymanski W. W., McMurry P. H., Roberts D. L., Pui D. Y. H. (2017). Characterization
of Nanosized Silica Size Standards. Aerosol
Sci. Technol..

[32] Park S., Choi Y. K., Kim S., Lee J., Im W. (2021). CHARMM-GUI
Membrane Builder for Lipid Nanoparticles with Ionizable Cationic Lipids
and PEGylated Lipids. J. Chem. Inf. Model..

[33] Cheng X., Jo S., Lee H. S., Klauda J. B., Im W. (2013). CHARMM-GUI Micelle
Builder for Pure/Mixed Micelle and Protein/Micelle Complex Systems. J. Chem. Inf. Model..

[34] Jo S., Kim T., Iyer V. G., Im W. (2008). CHARMM-GUI: A Web-Based Graphical
User Interface for CHARMM. J. Comput. Chem..

[35] Ovesný M., Křížek P., Borkovec J., Švindrych Z., Hagen G. M. (2014). ThunderSTORM: A
Comprehensive ImageJ Plug-in for PALM
and STORM Data Analysis and Super-Resolution Imaging. Bioinformatics.

[36] Davis J. L., Soetikno B., Song K. H., Zhang Y., Sun C., Zhang H. F. (2020). RainbowSTORM: An
Open-Source ImageJ Plug-in for Spectroscopic
Single-Molecule Localization Microscopy (SSMLM) Data Analysis and
Image Reconstruction. Bioinformatics.

[37] Souza P. C. T., Alessandri R., Barnoud J. (2021). Martini 3: a general
purpose force field for coarse-grained molecular dynamics. Nat. Methods.

[57] Pedersen K. B., Ingólfsson H.
I., Ramirez-Echemendia D.
P., Borges-Araújo L., Andreasen M. D., Empereur-mot C., Melcr J., Ozturk T. N., Bennett D. W. F., Kjolbye L. R. (2024). The Martini 3 Lipidome:
Expanded and Refined Parameters Improve Lipid Phase Behavior. ChemRxiv.

[38] Borges-Araújo L., Borges-Araújo A. C., Ozturk T. N., Ramirez-Echemendia D.
P., Fábián B., Carpenter T. S., Thallmair S., Barnoud J., Ingólfsson H.
I., Hummer G., Tieleman D. P., Marrink S. J., Souza P. C. T., Melo M. N. (2023). Martini
3 Coarse-Grained Force Field for Cholesterol. J. Chem. Theory Comput..

[39] Qi Y., Ingólfsson H. I., Cheng X., Lee J., Marrink S. J., Im W. (2015). CHARMM-GUI
Martini Maker for Coarse-Grained Simulations with the
Martini Force Field. J. Chem. Theory Comput..

[40] Emami F. S., Puddu V., Berry R. J., Varshney V., Patwardhan S. V., Perry C. C., Heinz H. (2014). Force Field
and a Surface Model Database
for Silica to Simulate Interfacial Properties in Atomic Resolution. Chem. Mater..

[41] Kim D., Zuidema J. M., Kang J., Pan Y., Wu L., Warther D., Arkles B., Sailor M. J. (2016). Facile Surface Modification
of Hydroxylated Silicon Nanostructures Using Heterocyclic Silanes. J. Am. Chem. Soc..

[42] Abraham M. J., Murtola T., Schulz R., Páll S., Smith J. C., Hess B., Lindah E. (2015). GROMACS: High Performance
Molecular Simulations through Multi-Level Parallelism from Laptops
to Supercomputers. SoftwareX.

[43] Coarse-Graining of Condensed Phase and Biomolecular Systems; CRC Press, 2008. 10.1201/9781420059564.

[44] Bussi G., Donadio D., Parrinello M. (2007). Canonical Sampling through Velocity
Rescaling. J. Chem. Phys..

[45] Bernetti M., Bussi G. (2020). Pressure Control Using Stochastic Cell Rescaling. J. Chem. Phys..

[46] Vögele M., Hummer G. (2016). Divergent
Diffusion Coefficients in Simulations of
Fluids and Lipid Membranes. J. Phys. Chem. B.

[47] Dusoswa S. A., Horrevorts S. K., Ambrosini M., Kalay H., Paauw N. J., Nieuwland R., Pegtel M. D., Würdinger T., Van Kooyk Y., Garcia-Vallejo J. J. (2019). Glycan Modification of Glioblastoma-Derived
Extracellular Vesicles Enhances Receptor-Mediated Targeting of Dendritic
Cells. J. Extracell. Vesicles.

[48] Fuller N. L., Benatti C. R., Rand R. P. (2003). Curvature
and Bending Constants for
Phosphatidylserine-Containing Membranes. Biophys.
J..

[49] Maggini L., Cabrera I., Ruiz-Carretero A., Prasetyanto E. A., Robinet E., De Cola L. (2016). Breakable Mesoporous
Silica Nanoparticles
for Targeted Drug Delivery. Nanoscale.

[50] Schade D. S., Shey L., Eaton R. P. (2020). Cholesterol
Review: A Metabolically
Important Molecule. Endocr. Pract..

[51] Nsairat H., Khater D., Sayed U., Odeh F., Al Bawab A., Alshaer W. (2022). Liposomes: Structure, Composition, Types, and Clinical
Applications. Heliyon.

[52] Skotland T., Sandvig K., Llorente A. (2017). Lipids in
Exosomes: Current Knowledge
and the Way Forward. Prog Lipid Res. Progress
In Lipid Research.

[53] Llorente A., Skotland T., Sylvänne T., Kauhanen D., Róg T., Orłowski A., Vattulainen I., Ekroos K., Sandvig K. (2013). Molecular
Lipidomics of Exosomes Released by PC-3 Prostate Cancer Cells. Biochim. Biophys. Acta, Protein Struct. Mol. Enzymol..

[54] National Center for Biotechnology Information PubChem. Compound Summary For CID 24261, Silicon Dioxide; National Center for Biotechnology Information. 2024.

[55] Takai C., Watanabe H., Asai T., Fuji M. (2012). Determine Apparent
Shell Density for Evaluation of Hollow Silica Nanoparticle. Colloids Surf., A.

[56] Yazdimamaghani M., Barber Z. B., Hadipour
Moghaddam S.
P., Ghandehari H. (2018). Influence
of Silica Nanoparticle Density and Flow Conditions on Sedimentation,
Cell Uptake, and Cytotoxicity. Mol. Pharmaceutics.

