# Supplementary material for: Rational Design of EV-Mimicking Nanoparticles with Polarity-Based Recognition Potential for Advanced Nanocarrier Development
Source: ACS Appl Nano Mater. 2025 Jun 23;8(26):13257–73. doi: 10.1021/acsanm.5c01459 (PMC12235584; doi:10.1021/acsanm.5c01459)
Supplement: Supplementary file 1 [file an5c01459_si_001.pdf]

Supporting Information

Rational Design of EV-Mimicking Nanoparticles with Polarity-Based Recognition Potential for Advanced Nanocarrier Development

Giada Rosso ‡[1], Stijn M. A. Van Veen ‡[2], María Sancho-Albero [3,¥], Giulia Tamboia [3,6,7], Charly Empereur-mot [4], Claudio Perego [4], Marije E. Kuipers [5], Bianca Dumontel [1], Alessandro Ajó [3], Esther N. M. Nolte-'t Hoen [5], Giovanni M. Pavan [1,4], Luisa De Cola [3,6], Lorenzo Albertazzi [2], Valentina Cauda [1*]*

[1] Department of Applied Science and Technology, Politecnico di Torino, Turin, Italy

[2] Institute of Complex Molecular Systems, Technische Universiteit Eindhoven, Eindhoven, The Netherlands

[3] Department of Biochemistry and Molecular Pharmacology, Istituto di Ricerche Farmacologiche Mario Negri IRCCS, Milan, Italy

[4] Department of Innovative Technologies, Institute for Mechanical Engineering and Materials Technologies, University of Applied Science and Arts of Southern Switzerland, Lugano, Switzerland

[5] Department of Biomolecular Health Sciences, Utrecht University, Utrecht, The Netherlands

[6] Department of Pharmaceutical Science, DISFARM, Università degli Studi di Milano, 20133 Milan, Italy

[7] Department of Chemistry, Biology and Biotechnology, Università di Perugia, 06123 Perugia, Italy

‡ These authors have equally contributed to the work.

[¥] Present address: Instituto de Nanociencia y Materiales de Aragón (INMA), CSIC-Universidad de Zaragoza, Campus Rio Ebro, Edificio I+D+I, C/Poeta Mariano Esquillor, s/n, 50018, Zaragoza, Spain. Department of Chemical and Environmental Engineering, Institute of Nanoscience and Materials of Aragon, Universidad de Zaragoza, Zaragoza, Spain. Instituto de Investigación Sanitaria de Aragón (IIS Aragón), Avda. San Juan Bosco, 13, 50009, Zaragoza Spain. Networking Research Center in Biomaterials, Bioengineering and Nanomedicine (CIBERBBN), Insituto de Salud Carlos III, 28029, Madrid, Spain.

*Corresponding authors:

Prof. Valentina Cauda phone +39 011 0907389, e-mail: valentina.cauda@polito.it

Prof. Lorenzo Albertazzi phone +31 40 247 5997, e-mail: l.albertazzi@tue.nl

Table S1. Mass percentage of different lipid species present in PC3-derived EVs, as reported by Ferreri et al [25].

Lipid Families in PC3dEVs		Mass percentage	SEM
**Chol**	Cholesterol	12.6%	2.2%
**SM**	Sphingomyelins	20.2%	11.8%
**PE**	Phosphatidyl Ethanolamine	16.1%	4.9%
**PS**	Phosphatidyl Serine	26.1%	7.4%
**PC**	Phosphatidyl Choline	25.0%	9.6%

Table S2. Mass percentage of the different fatty acids present in PC3-derived EVs, as reported by Ferreri et al [25].

Fatty acids in PC3dEVs		Mass percentage	SEM
**Saturated Fatty Acids (SFA) Tot= 50.04 ± 3.23 %**	C14:0	4.32%	1.12%
	C16:0	33.30%	1.25%
	C18:0	12.42%	0.86%
**MonoUnsaturated Fatty Acids (MUFA) Tot= 42.37 ± 7.19 %**	6trans-C16:1	0.09%	0.03%
	6cis-C16:1 n-10	6.02%	1.53%
	9cis-C16:1 n-7	1.12%	0.30%
	9trans-C18:1	0.18%	0.04%
	8cis-C18:1 n-10	2.85%	0.66%
	9cis-C18:1 n-9	30.88%	4.52%
	11cis-C18:1 n-7	1.22%	0.11%
**PolyUnsaturated Fatty Acids (PUFA) Tot= 7.59 ± 1.30 %**	5cis,8cis-C18:2 n-10	0.26%	0.04%
	mono-trans C18:2 n-6	0.15%	0.03%
	C18:2 n-6	3.41%	0.52%
	C20:3 n-6	0.49%	0.08%
	C20:4 n-6	0.73%	0.17%
	mono-trans C20:4	0.04%	0.02%
	C20:5 n-3	0.28%	0.04%

C22:5 n-3	0.54%	0.14%
C22:6 n-3	1.69%	0.27%

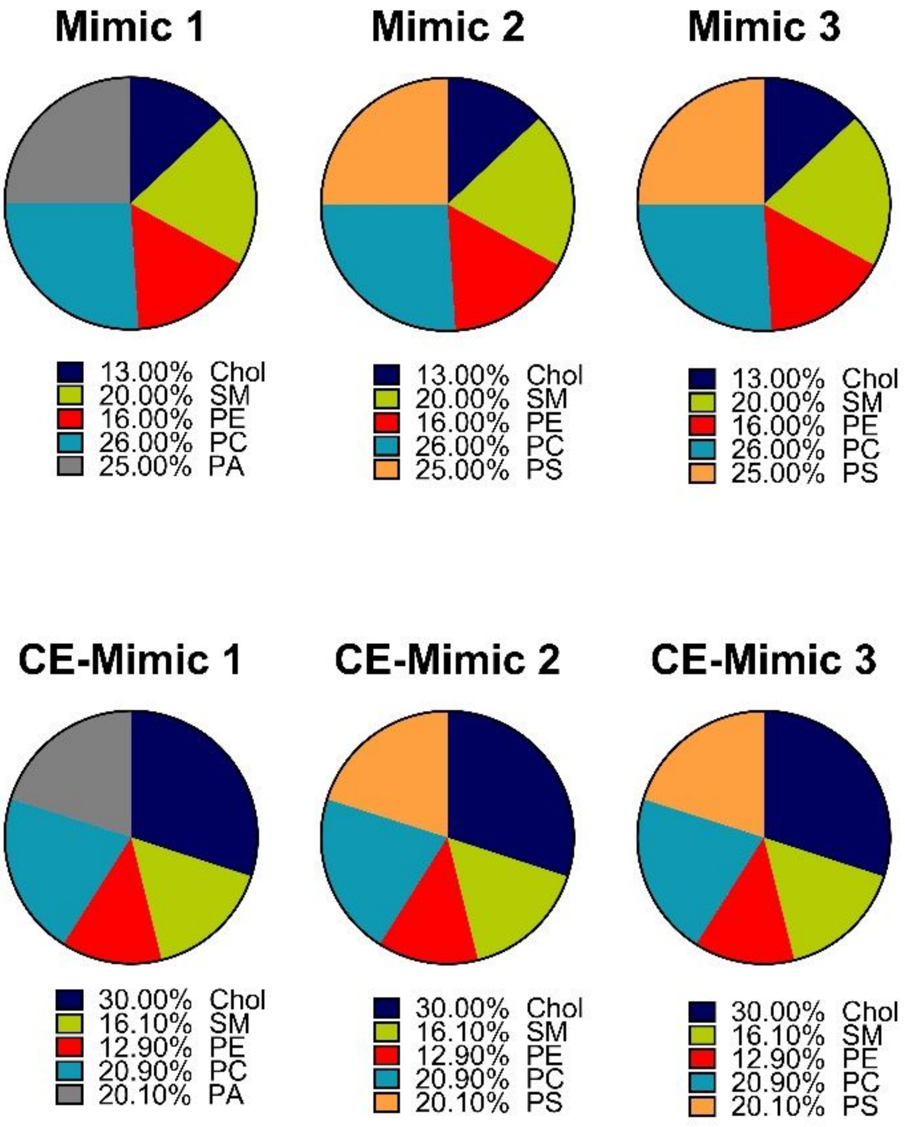

Figure S1. Composition of the Cholesterol-Enhanced Mimic formulations, compared to the first version of Mimic formulations

Nanocapsules synthesis and characterization

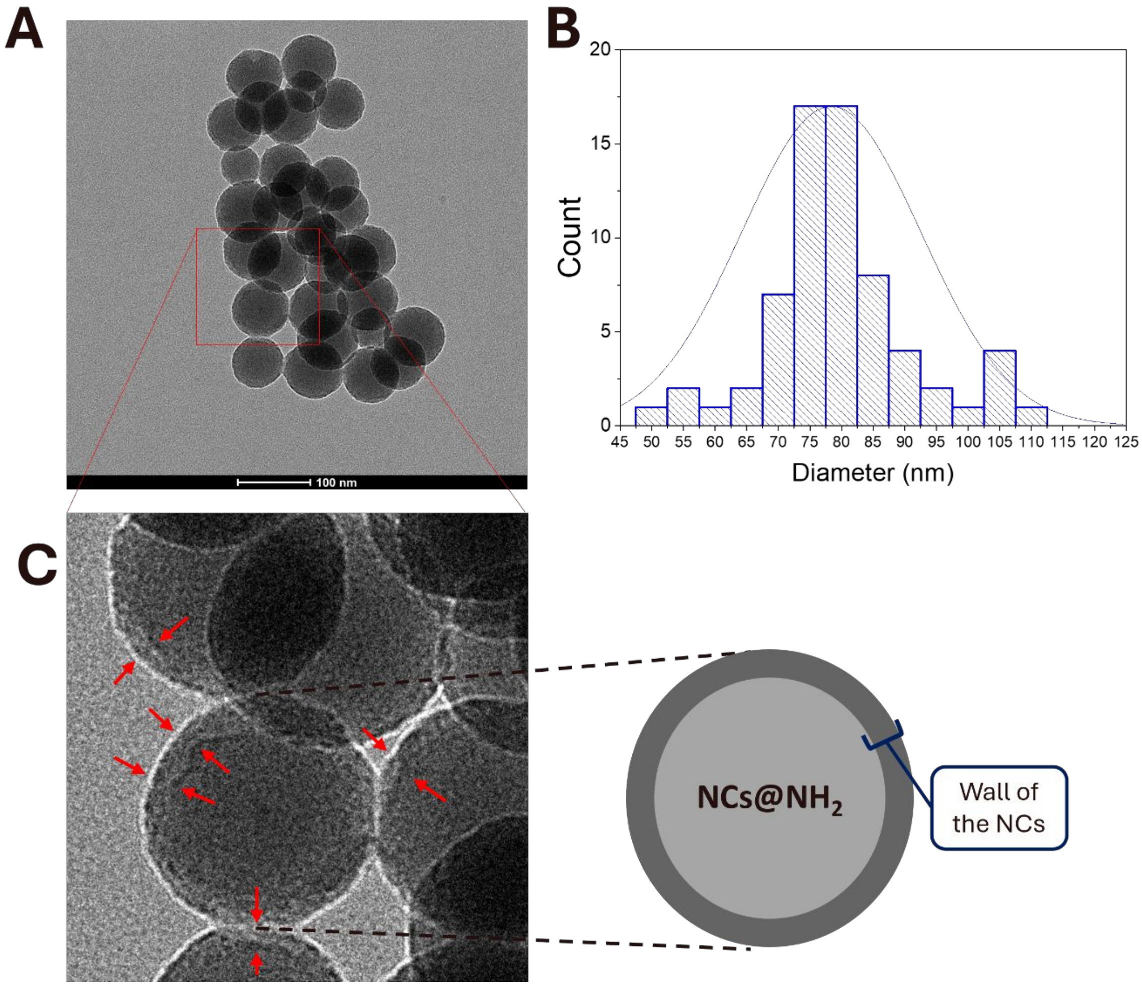

Figure S2. A) TEM images of uncoated NCs; B) Size distribution of NCs, as calculated from TEM images (average equals to 79.4 ± 11.6 nm) C) close-up view of uncoated NCs, where the wall of the NCs was highlighted with red arrows.

FT-IR spectra (**Figure**) revealed the presence of disulfide bonds incorporated within the silica framework of the NCs. The characteristic absorption bands of the methyl groups connected to the disulfide unit occur at 1456^{-1} and 1354 cm^{-1} attributed to the -CH_2 wagging and deformation vibration. Additionally, several peaks originating from the positively-charged silacyclooctane are clearly observable (1523 cm $^{-1}$, 1626 cm^{-1}, and 2600-3650 cm^{-1} of the NH_2 bands), confirming

again the amine functionalization of the NCs. Finally, by TGA it was possible to quantify the amount of S-S groups present in the naked NPs and in the NCs@NH_2, at approximately 30 % of the sample mass (see weight loss n˚1 in Figure 2E). In the NCs@NH_2 an additional weight decay at a higher temperature was observed corresponding with a mass loss of 15 % (weight loss n˚2) which can be attributed to the presence of the silacyclooctane responsible for the amine groups. These two analyses confirm clearly the successful functionalization of the NCs with amino-groups.

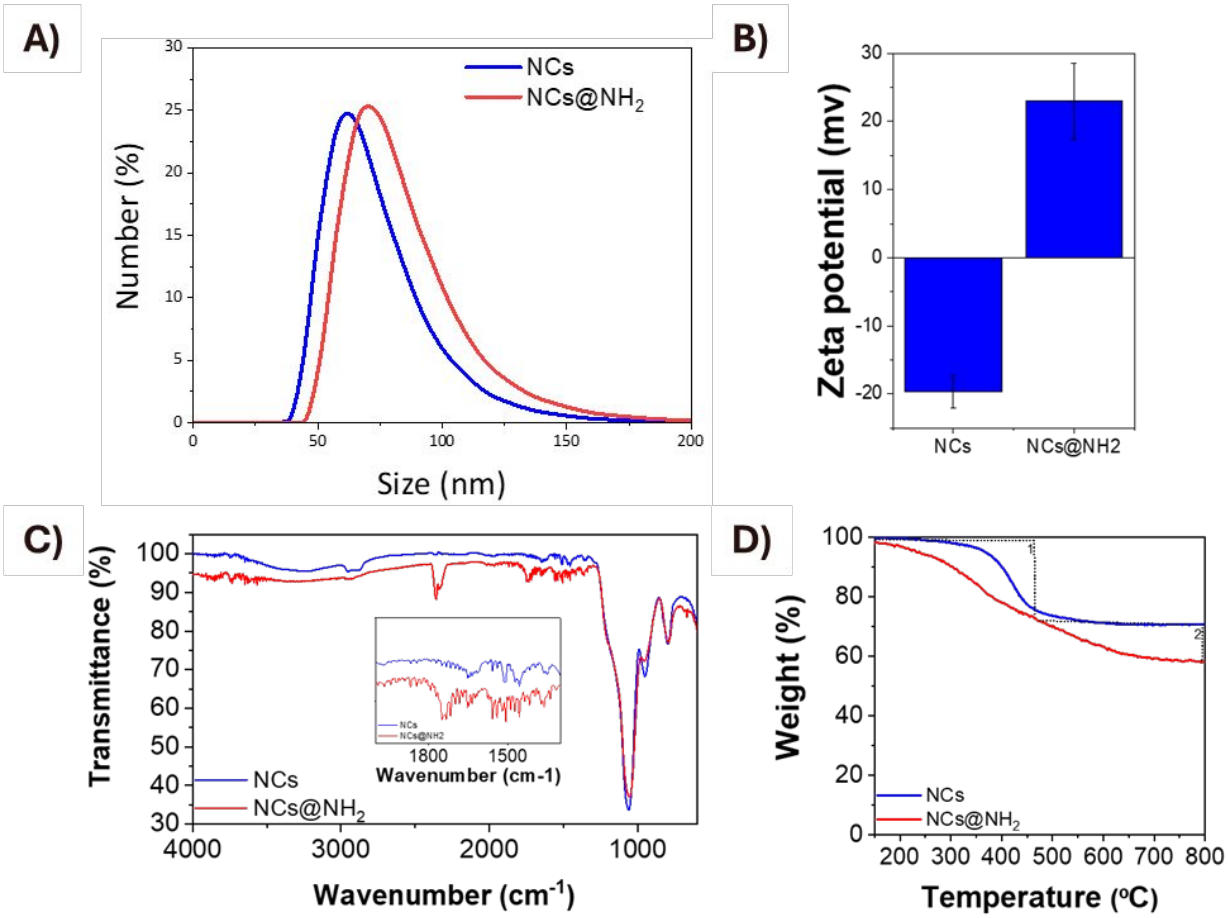

Figure S3. Characterization of NCs (blue lines) and NCs@NH2 (red lines). A) Hydrodynamic diameter and size distribution of naked NCs and NCs@NH2 in water obtained by DLS. B) Zeta potential values of the NCs before and after functionalization with the positively charged silacyclooctane C) FTIR transmission spectra of pristine (NCs) and functionalized organosilica nanocapsules (NCs@NH_2). D) TGA analysis of the nanocapsules.

Optimization of lipid/NCs ratio

Table S3. Estimation of the weight of a silica NCs, considered as a perfect hollow sphere of a given diameter, wall thickness equal to 3.68 nm and 1.87 g/m^3 of density.

	**Outer diameter (nm):**	**50**	**55**	**60**	**65**	**70**	**75**	**80**	**85**	**90**	**95**	**100**	**105**	**110**	**AVG**
**NCs weight (g)**	**Inner diameter (nm)**	42.6	47.6	52.6	57.6	62.6	67.6	72.6	77.6	82.6	87.6	92.6	97.6	102.6	72.6
	**NCs volume (nm^3)**	2.5 E-23	3.1 E-23	3.7 E-23	4.4 E-23	5.1 E-23	5.9 E-23	6.7 E-23	7.7 E-23	8.6 E-23	9.6 E-23	1.1 E-22	1.2 E-22	1.3 E-22	7.1 E-23
	**NCs weight (g)**	4.6 E-17	5.7 E-17	6.9 E-17	8.1 E-17	9.5 E-17	1.1 E-16	1.3 E-16	1.4 E-16	1.6 E-16	1.8 E-16	2.0 E-16	2.2 E-16	2.4 E-16	1.3 E-16

Table S4. Estimation of the weight of a lipid bilayer around a NCs of a given diameter.

	**Outer diameter (nm):**	**50**	**55**	**60**	**65**	**70**	**75**	**80**	**85**	**90**	**95**	**100**	**105**	**110**
**Lipids weight (g)**	3C	2.7E-17	3.2E-17	3.6E-17	4.1E-17	4.6E-17	5.1E-17	5.6E-17	6.2E-17	6.8E-17	7.4E-17	8.1E-17	8.8E-17	9.5E-17
	MIMIC 1	3.2E-17	3.7E-17	4.2E-17	4.7E-17	5.3E-17	5.9E-17	6.6E-17	7.3E-17	8.0E-17	8.7E-17	9.5E-17	1.0E-16	1.1E-16
	CE-MIMIC 1	2.7E-17	3.2E-17	3.6E-17	4.1E-17	4.5E-17	5.1E-17	5.6E-17	6.2E-17	6.8E-17	7.4E-17	8.1E-17	8.7E-17	9.4E-17
	MIMC 2	3.2E-17	3.7E-17	3.9E-17	4.8E-17	5.4E-17	6.0E-17	6.6E-17	7.3E-17	8.0E-17	8.8E-17	9.5E-17	1.0E-16	1.1E-16
	CE-MIMIC 2	2.8E-17	3.2E-17	3.6E-17	4.1E-17	4.6E-17	5.1E-17	5.7E-17	6.2E-17	6.8E-17	7.5E-17	8.1E-17	8.8E-17	9.5E-17
	MIMIC 3	3.1E-17	3.6E-17	4.1E-17	4.6E-17	5.2E-17	5.8E-17	6.4E-17	7.0E-17	7.7E-17	8.4E-17	9.2E-17	1.0E-16	1.1E-16
	CE-MIMIC 3	2.7E-17	3.1E-17	3.5E-17	4.0E-17	4.5E-17	5.0E-17	5.5E-17	6.1E-17	6.6E-17	7.3E-17	7.9E-17	8.6E-17	9.2E-17

Table S5. Estimation of the NCs/lipids mass ratio, considering NCs as a perfect hollow sphere of a given diameter, wall thickness equal to 3.68 nm and 1.87 g/m³ of density.

	Outer NCs diameter (nm):	50	55	60	65	70	75	80	85	90	95	100	105	110
NCs/Lipids weight ratio	3C	1.7	1.8	1.9	2.0	2.1	2.2	2.2	2.3	2.4	2.4	2.5	2.5	2.6
	MIMIC 1	1.5	1.5	1.6	1.7	1.8	1.9	1.9	2.0	2.0	2.1	2.1	2.2	2.2
	CE-MIMIC 1	1.7	1.8	1.9	2.0	2.1	2.2	2.2	2.3	2.4	2.4	2.5	2.5	2.6
	MIMC 2	1.4	1.5	1.8	1.7	1.8	1.8	1.9	2.0	2.0	2.1	2.1	2.1	2.2
	CE-MIMIC 2	1.7	1.8	1.9	2.0	2.1	2.2	2.2	2.3	2.4	2.4	2.5	2.5	2.6
	MIMIC 3	1.5	1.6	1.7	1.8	1.8	1.9	2.0	2.0	2.1	2.1	2.2	2.2	2.3
	CE-MIMIC 3	1.7	1.8	2.0	2.0	2.1	2.2	2.3	2.4	2.4	2.5	2.5	2.6	2.6

Table S6. Weighting of the NCs/lipids mass ratio, considering the distribution of NCs obtained from TEM images. Silica density was set to 1.87 g/m³.

**Bin Start:**		**47.5**	**52.5**	**57.5**	**62.5**	**67.5**	**72.5**	**77.5**	**82.5**	**87.5**	**92.5**	**97.5**	**102.5**	**107.5**	
**Bin end:**		**52.5**	**57.5**	**62.5**	**67.5**	**72.5**	**77.5**	**82.5**	**87.5**	**92.5**	**97.5**	**102.5**	**107.5**	**112.5**	
**frequency percentage:**		0%	1%	3%	1%	3%	10%	25%	25%	12%	6%	3%	1%	6%	**Sum**
**Centered Bins**	3C	0.00	0.03	0.06	0.03	0.06	0.23	0.57	0.59	0.28	0.15	0.07	0.04	0.15	2.25
	MIMIC 1	0.00	0.02	0.05	0.03	0.05	0.19	0.49	0.50	0.24	0.12	0.06	0.03	0.13	1.92
	CE-MIMIC 1	0.00	0.03	0.06	0.03	0.06	0.23	0.57	0.59	0.28	0.15	0.07	0.04	0.15	2.26
	MIMC 2	0.00	0.02	0.05	0.03	0.05	0.19	0.48	0.50	0.24	0.12	0.06	0.03	0.13	1.91
	CE-MIMIC 2	0.00	0.03	0.06	0.03	0.06	0.23	0.57	0.58	0.28	0.14	0.07	0.04	0.15	2.24

	MIMIC 3	0.00	0.02	0.05	0.03	0.05	0.20	0.50	0.52	0.25	0.13	0.07	0.03	0.14	1.98
	CE-MIMIC 3	0.00	0.03	0.06	0.03	0.06	0.23	0.58	0.60	0.29	0.15	0.08	0.04	0.16	2.30

Table S7. Description of the molecular content and characteristics of the systems simulated in this study. PSM: Sphyngomyelin 16:0. DSPE-PEG: 1,2-Distearoyl-sn-glycero-3-phosphoethanolamine

	Molecular content							Solvent			Surface lattice sites	System size (nm)
	PSM	DSPE-PEG	DIPE	DOPC	DPPS	DOPA	CHOL	W	Na+	Cl-		
**Mimic 1 Free**	143	29	0	166	0	173	485	32989	174	0	0	14.8 x 14.8 x 22.7
**Mimic 1 Supported**	143	29	0	166	0	173	485	29563	87	513	600	14.4 x 14.4 x 23.5
**Mimic 2 Free**	144	29	0	167	167	0	489	31885	168	0	0	14.4 x 14.4 x 23.4
**Mimic 2 Supported**	144	29	0	167	167	0	489	28597	84	492	576	14.1 x 14.1 x 23.9
**Mimic 3 Free**	139	18	39	162	162	0	475	32948	163	0	0	14.6 x 14.6 x 23.4
**Mimic 3 Supported**	139	18	39	162	162	0	475	29654	82	495	576	14.2 x 14.2 x 24.2

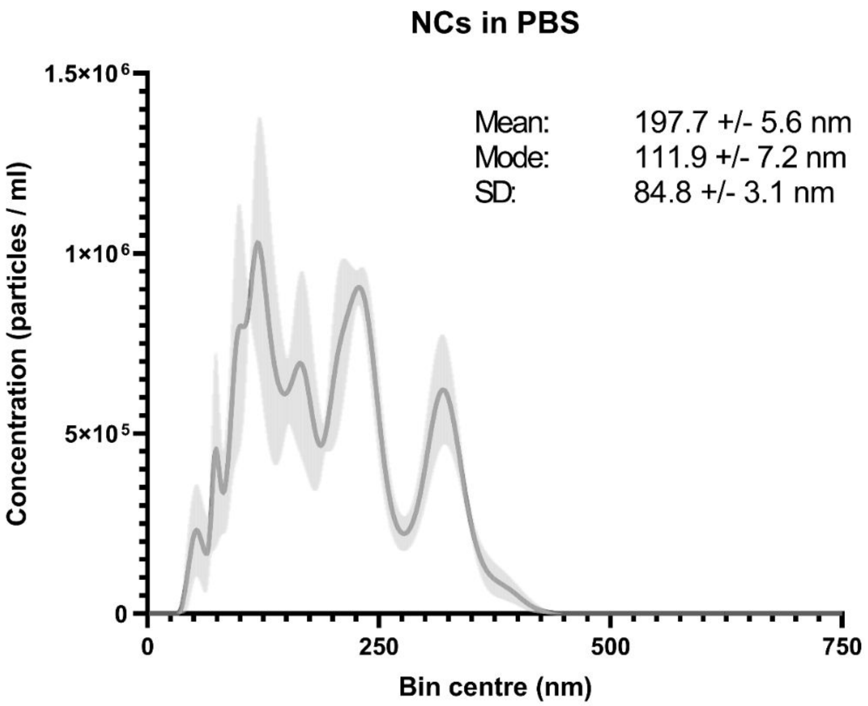

Figure S4. Nanopaticle tracking analysis (NTA) of uncoated NCs in PBS

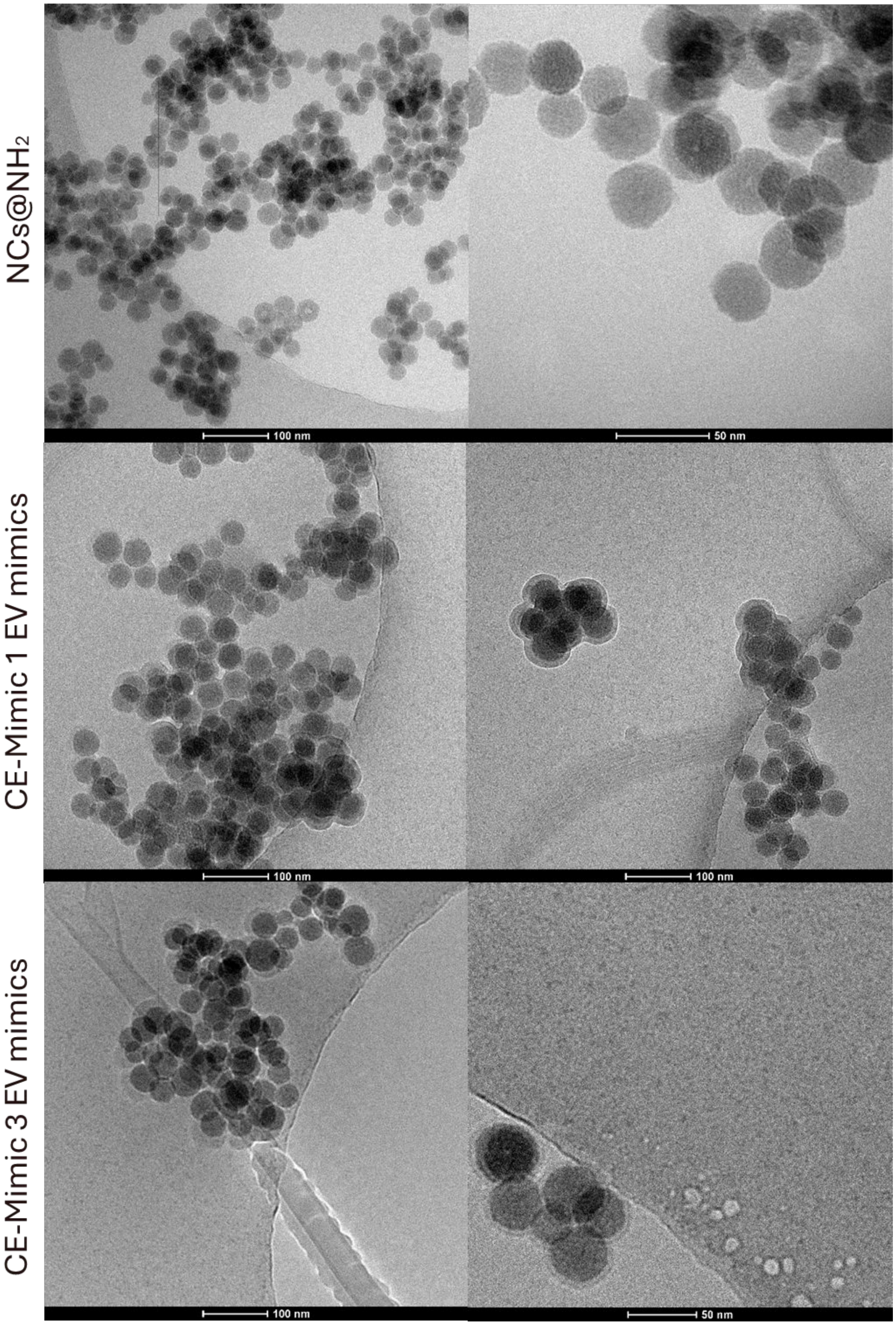

Figure S5. Cryo-TEM images of uncoated NCs@NH_2, in comparison to lipid-coated NCs@NH_2, namely CE-Mimic1 EV mimics and CE-Mimic3 EV mimics. EV mimics appear nanoparticles surrounded by a single lipid bilayer.

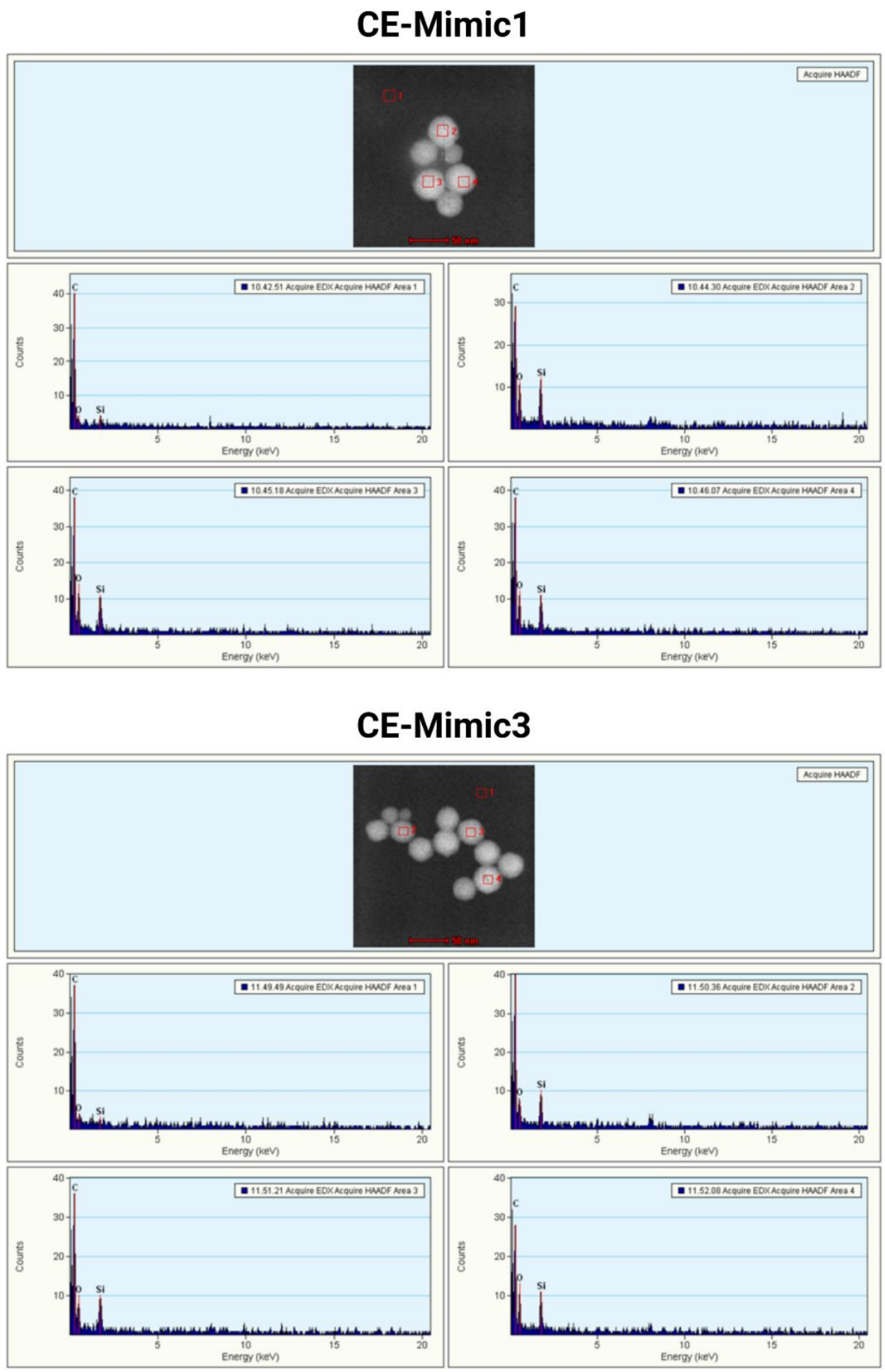

Figure S6. Scanning-Transmission Electron Microscopy (STEM) images of EV Mimics (CE-Mimic1 and CE-Mimic3) and relative X-ray Energy Dispersive Spectra (EDS), measured to analyze the chemical composition of the materials and corroborate the presence of Si in the EV Mimics.

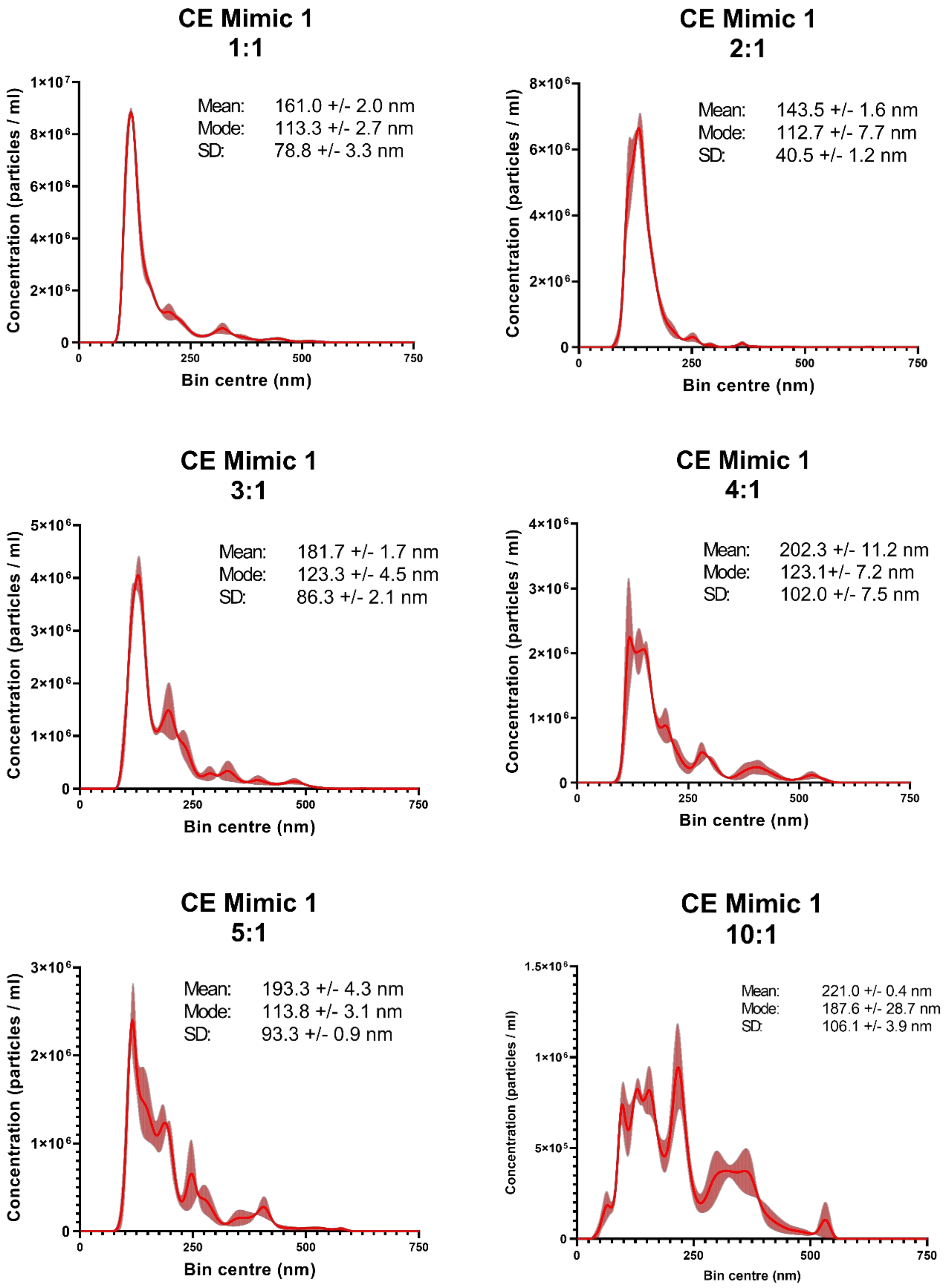

Figure S7. Nano tracking analysis (NTA) of NCs coated with different ratios of CE Mimic 1 formulation in PBS.

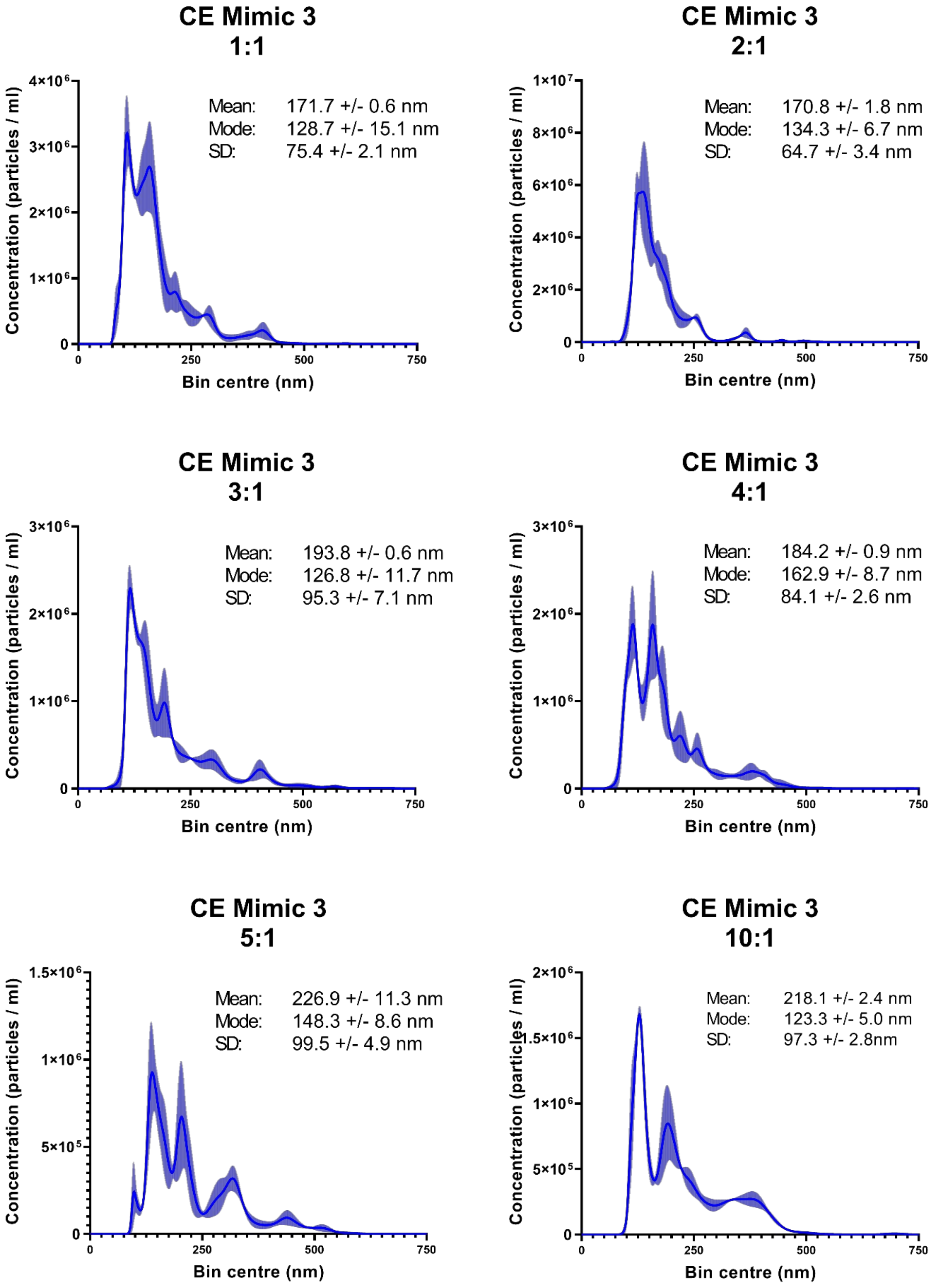

Figure S8. Nano tracking analysis (NTA) of NCs coated with different ratios of CE Mimic 3 formulation in PBS.

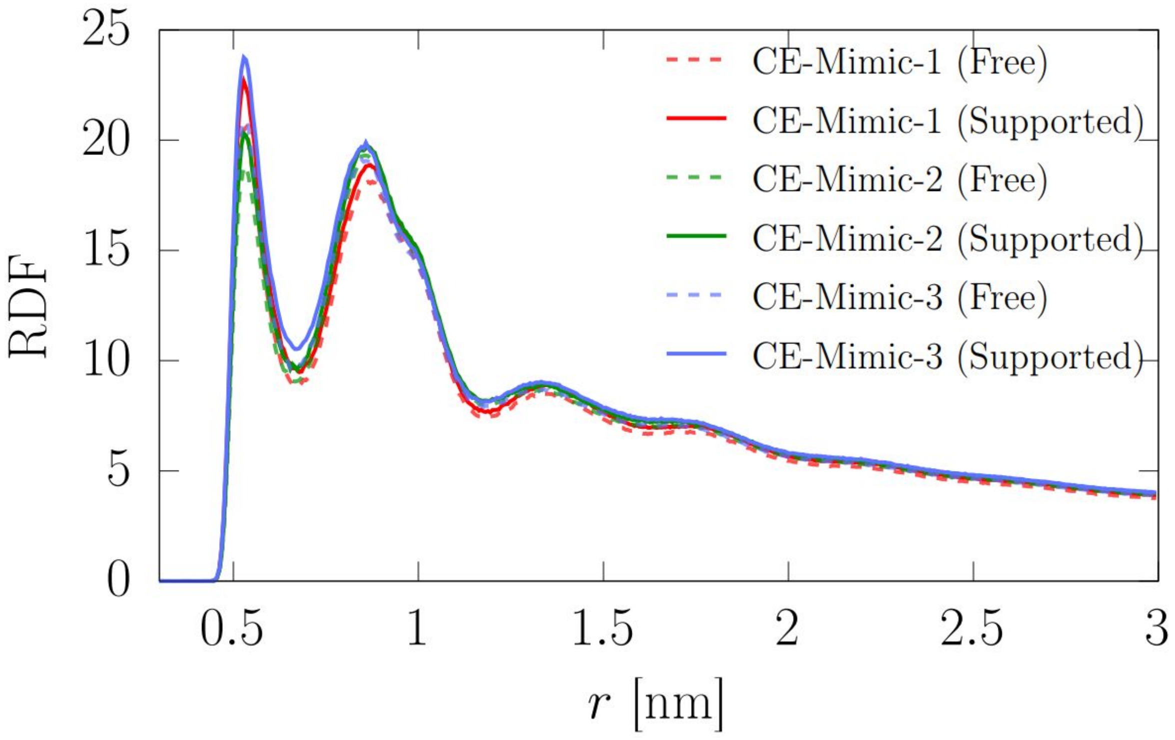

Figure S9. Radial distribution functions computed for all lipids in the upper leaflet of the simulated bilayers for CE-Mimic 1, 2 and 3, for the membrane in solvent (Free: dashed lines) or non-covalently bound to a functionalized organosilica surface (Supported: plain lines).

High-resolution single-particle analysis

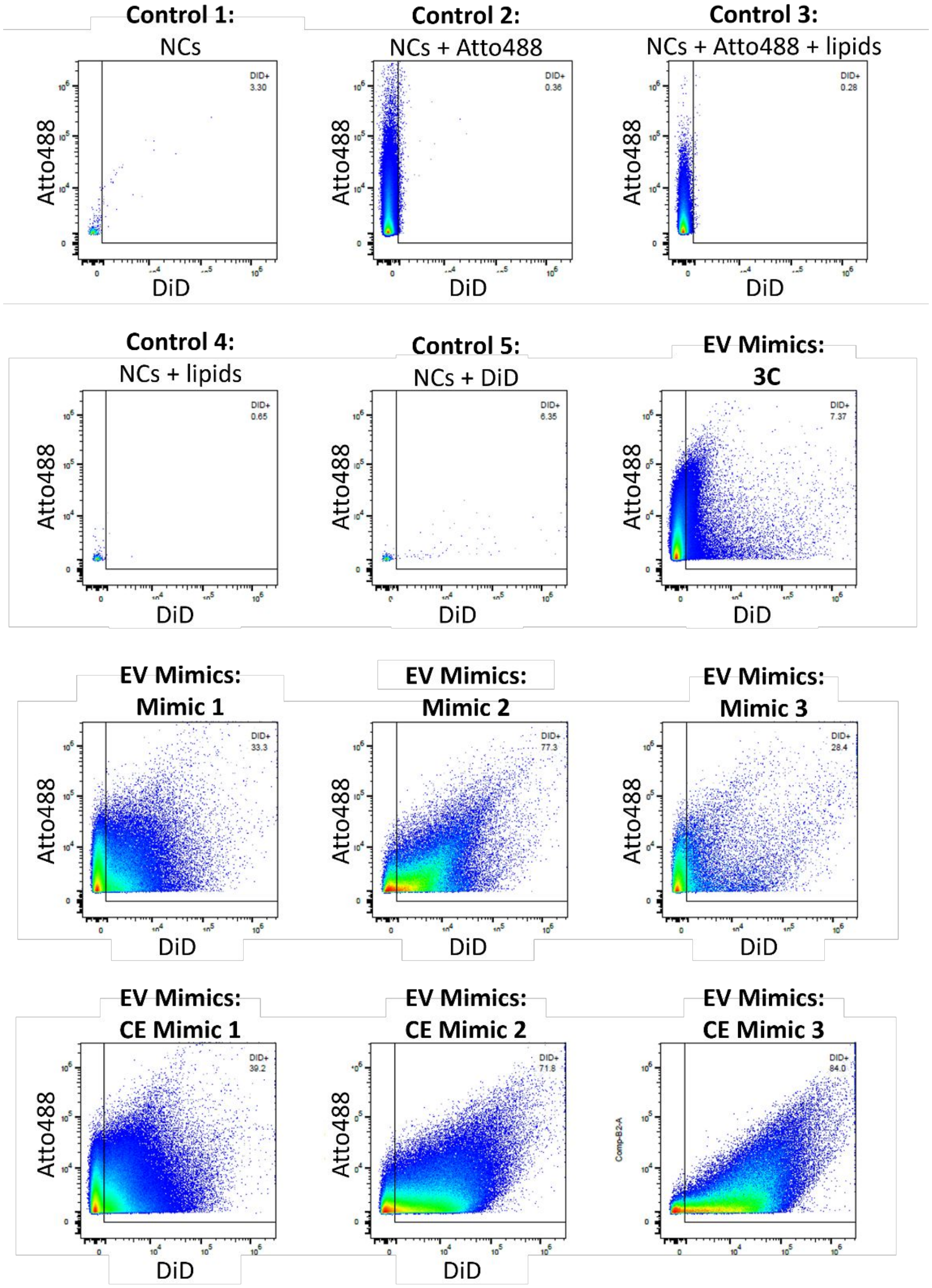

Figure S10. High resolution flow cytometry analysis of EV mimics: dot plots of control samples and EV mimics. In EV mimics samples, NCs were labelled with Atto488 (Y axis) and lipids were stained with the lipophilic dye DiD (X axis).

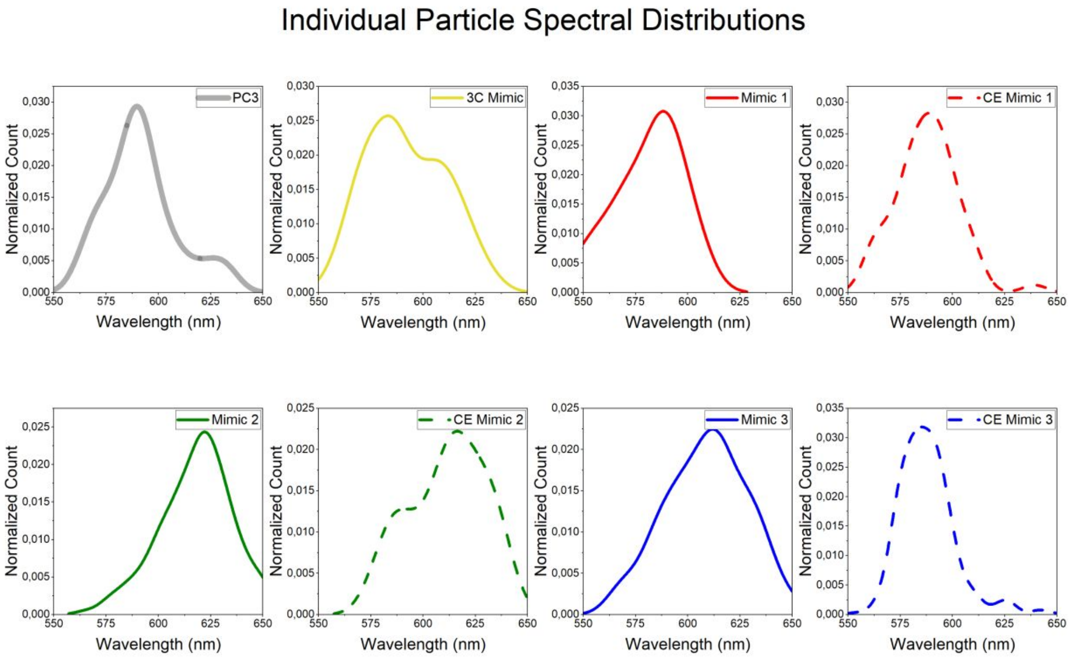

Figure S11. Distributions of fitted peak wavelengths for all localizations belonging to a single particle in the population.

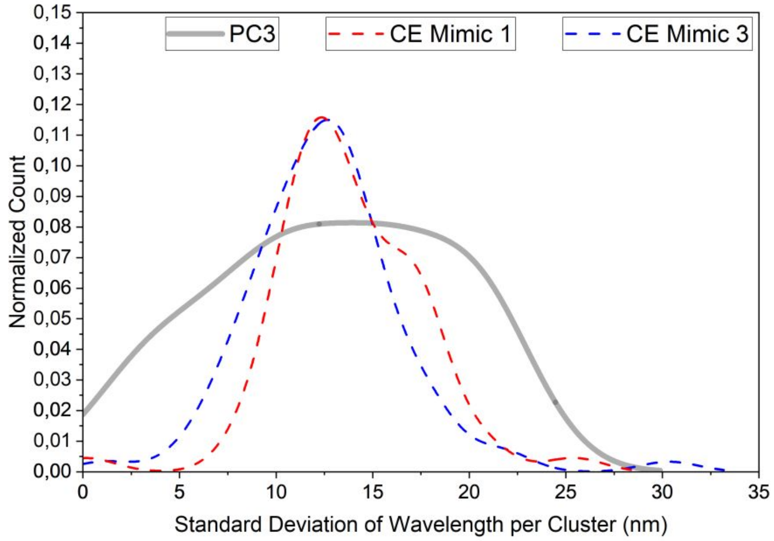

Figure S12. Distributions of the standard deviation calculated from all localizations per particle, for all particles measured belonging to that sample. A larger standard deviation means that particle is more heterogeneous.

Cell internalization of NCs and EV Mimics

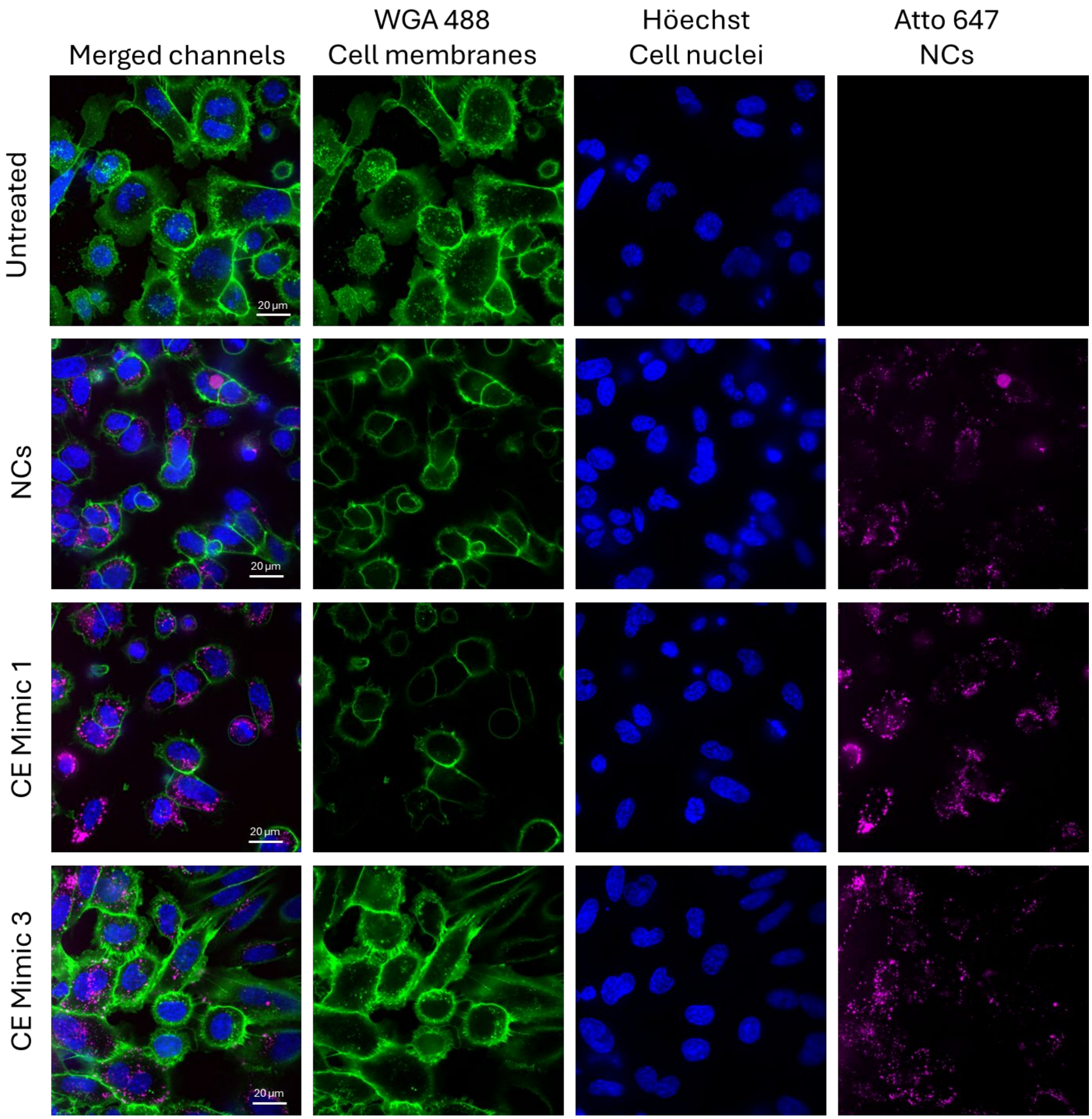

Figure S13. Qualitative microscopy images of the internalization test performed on Saos2 cells. This cell line was chosen since the final target of the EV Mimics is the bone tissue, showing the PC3-derived EVs an organotropism towards the bones. The images were taken after 24 hours of incubation of the NCs or the best performing EV Mimics. The NCs were labelled with Atto 647 prior and eventually coated with the EV-mimicking formulations. From these images it is clearly visible that both the NCs and the EV Mimics are massively internalized in the Saos2 cells.

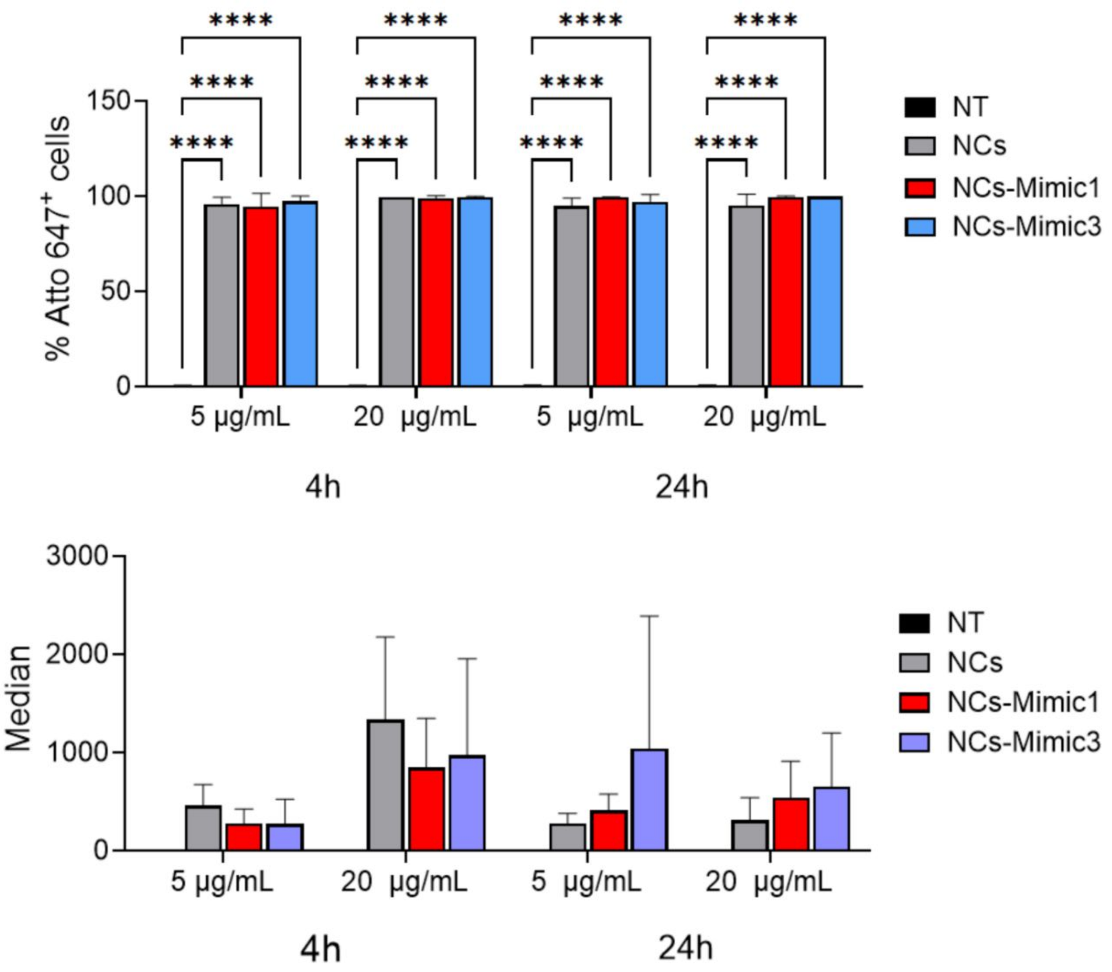

Figure S14. The uptake of the EV Mimics by Saos2 cells was as well evaluated through flow cytometry. The obtained data confirm that the nanoconstructs were internalized by the large majority of the cells, even in the first time step (4h). Since no significant difference was evidenced in the percentage of positive events, being all the samples being internalized by substantially 100% of the cells, the median of the signal's intensity is also reported. This second plot shows how NCs are internalized more than EV Mimics in the first 4h, a really quick internalization that can be explained considering that NCs are positively charged and thus can rapidly interact with the negatively charged membrane of the cells. However, at 24h post administration, the signal from the EV Mimics is stronger than the one of uncoated NCs, higher in the case of CE Mimic 3 sample.
